# Craniofacial syndromes and class III phenotype: common genotype fingerprints? A scoping review and meta-analysis

**DOI:** 10.1038/s41390-023-02907-5

**Published:** 2024-02-12

**Authors:** Maria Cristina Faria-Teixeira, Cristina Tordera, Francisco Salvado e Silva, António Vaz-Carneiro, Alejandro Iglesias-Linares

**Affiliations:** 1 Complutense University of Madrid, School of Dentistry, 28040 Madrid, Spain; 2 University of Lisbon, School of Medicine, University Clinic of Stomatology, 1200 Lisbon, Portugal; 3 Institute for Evidence Based Healthcare, 1649-028 Lisboa, Portugal; 4 BIOCRAN (Craniofacial Biology) Research Group, Complutense University, 28040 Madrid, Spain

## Abstract

**Abstract:**

Skeletal Class III (SCIII) is among the most challenging craniofacial dysmorphologies to treat. There is, however, a knowledge gap regarding which syndromes share this clinical phenotype. The aims of this study were to: (i) identify the syndromes affected by the SCIII phenotype; (ii) clarify the involvement of maxillary and/or mandibular structures; (iii) explore shared genetic/molecular mechanisms. A two-step strategy was designed: [Step#1] OMIM, MHDD, HPO, GeneReviews and MedGen databases were explored; [Step#2]: Syndromic conditions indexed in [Step#1] were explored in Medline, Pubmed, Scopus, Cochrane Library, WOS and OpenGrey. Eligibility criteria were defined. Individual studies were assessed for risk of bias using the New Ottawa Scale. For quantitative analysis, a meta-analysis was conducted. This scoping review is a hypothesis-generating research. Twenty-two studies met the eligibility criteria. Eight syndromes affected by the SCIII were targeted: Apert syndrome, Crouzon syndrome, achondroplasia, X-linked hypohidrotic ectodermal dysplasia (XLED), tricho-dento-osseous syndrome, cleidocranial dysplasia, Klinefelter and Down syndromes. Despite heterogeneity between studies [*p* < 0.05], overall effects showed that midface components were affected in Apert and Down Syndromes, lower face in Klinefelter Syndrome and midface and lower face components in XLED. Our review provides new evidence on the craniofacial characteristics of genetically confirmed syndromes exhibiting the SCIII phenotype. Four major regulatory pathways might have a modulatory effect on this phenotype.

**Impact:**

What does this review add to the existing literature?

- To date, there is no literature exploring which particular syndromes exhibit mandibular prognathism as a common trait.
- Through this research, it was possibly to identify the particular syndromes that share the skeletal Class III phenotype (mandibular prognathism) as a common trait highlighting the common genetic and molecular pathways between different syndromes acknowledging their impact in craniofacial development.

## Introduction

The skeletal class III (SCIII) malocclusion phenotype represents a heterogeneous cluster of craniofacial anomalies characterized by an anterior position of the mandible relative to the cranial base (mandibular prognathism), a posterior position of the maxilla (maxillary hypoplasia) or a combination of both.^1–3^

Among the different types of sagittal skeletal discrepancies, SCIII is the type of malocclusion whose genetic mechanisms have been studied in most detail.^4,5^ Both environmental and genetic factors influence mandibular prognathism, although little is known about their interactions. Apprehending their impact however, could enhance the clinician’s ability to treat this malocclusion successfully.

SCIII malocclusion can occur as an isolated trait or as a clinical feature within particular craniofacial syndromes.^6,7^ The SCIII phenotype has been linked to the upregulation of specific molecular pathways involved in bone and cartilage development, suggesting its possible involvement in mandibular size discrepancy:^8,9^ more specifically, the fibroblast growth factor (FGFR), hedgehog (HH) and wingless (WNT) signaling pathways, and the transforming growth factor beta (TGF-β) signaling pathway, which includes the bone morphogenic proteins (BMPs) and activins.^9–13^ Variations in the coding sequence of these key regulatory genes and/ environmental factors often interfere with craniofacial development in a predictable way.^12^

Therefore, the SCIII phenotype linked to midface hypoplasia is commonly found in syndromes associated with FGFR mutations, such as Apert and Crouzon syndromes,^14^ which share a mutation in the fibroblast growth receptor 2 (FGFR2) gene.^14^ Achondroplasia is another FGFR-related syndrome, associated with FGFR3 mutations, and typically presents a SCIII phenotype related simultaneously to midface hypoplasia and mandibular prognathism.^1,15^ Disruptions in TGF-β and BMP signaling have been related to different syndromes exhibiting the SCIII phenotype. The signaling equilibrium can be disrupted by mutations in the EDA gene,^16–18^ frequently observed in X-linked hypohidrotic ectodermal dysplasia (XLHED), whose craniofacial phenotype involves maxillary retrognathia and hypoplasia as well as mandibular prognathism;^19,20^ Furthermore, DLX3^21^ and RUNX2^22^ mutations have been associated with tricho-dento-osseous syndrome (TDO)^21^ and cleidocranial dysplasia (CCD),^22^ respectively, interfering with important osteogenic regulatory factors^23–25^ and linked to a SCIII phenotype related to midface hypoplasia.^21,22^ The SCIII phenotype also occurs in aneuploidy disorders such as Down syndrome (DS)^26–30^ and Klinefelter syndrome (KS).^31–33^ Dysregulated WNT signaling has been related to KS.^34^ DS patients on the other hand, typically exhibit SCIII related to maxillary hypoplasia,^26–30^ which may be associated with unregulated SHH signaling.^35^

Hence, the SCIII phenotype can manifest as a particular clinical trait in several genetically well-categorized syndromes as deriving either from mandibular prognathism, maxillary hypoplasia, or a combination of both. Nevertheless, there is a critical knowledge gap with (i) inaccurate descriptions of the facial phenotype as a result of interchangeable use of the terms “mandibular prognathism”, “maxillary hypoplasia” and “SCIII malocclusion” in facial descriptions of several craniofacial syndromes, and (ii) a failure to use a precise, standardized cephalometric technique to analyze craniofacial skeletal features.

To address this knowledge gap, a scoping review methodology was adopted.^36–38^ The aim of the present scoping review was, firstly, to critically identify which syndromes are specifically affected by the SCIII phenotype as a clinically identifiable feature, and secondly, whether the maxillary and/or mandibular structure is involved when compared with non-syndromic subjects. Thirdly, the present scoping review may serve as a hypothesis-generating research, analyzing the shared chromosomal loci involved and/or common genetic mechanisms and molecular pathways that may be involved in the occurrence of syndromes with the SCIII phenotype. Finally, this review aims to facilitate decision-making and to promote interdisciplinary interaction among clinicians involved in the diagnosis and therapeutic guidance of syndromic patients.

## Materials and methods

### Protocol registration and guidelines

The methods and reporting of this scoping review followed the Preferred Reporting Items for Systematic Reviews and Meta-Analysis (PRISMA) extension for scoping reviews^39,40^ and of the Center for Reviews and Dissemination (CRD).^41^ The methods were developed following the guidance of the Cochrane Collaboration.^42,43^ The review protocol was pre-registered in Open Science Frame Work and is available at https://osf.io/9pa87/.

### Search strategy and sources of information

The search strategy used in the present scoping review was designed in a two-step protocol:

[*Step 1#*] In the first stage, the Online Mendelian Inheritance in Man Database (OMIM), and the MalaCards Human Disease Database (MHDD), Human Phenotype Ontology (HPO), GeneReviews and MedGen databases were explored, using a specific search string to identify all potential syndromic conditions with the SCIII phenotype (Supplementary File 1).

[*Step 2#*]: The indexed and categorized syndromic conditions from [*Step 1#]* were used for the second phase: a systematic search conducted in Medline, Pubmed, Scopus, Cochrane Library, Web of Science, together with a manual search up to May 2023. The Gray Open literature database was also searched, using a list of appropriate databases taken from the CADTH online resource.^44^

The complete search strategy is available in Supplementary File 1.

### Eligibility criteria for studies to be considered

[Step 1#]: *Electronic genetic databases* were explored (Supplementary File 1) to identify all available registered syndromic conditions that met two criteria: (i) SCIII phenotype/malocclusion as one of the characteristics of the clinical syndrome; (ii) available genomic information with affected locus/loci or genes associated with the particular syndromic condition.

[Step 2#]: In the second phase of this scoping review (Supplementary File 2), the eligibility criteria were defined according to the PICOS format: *(P) participants*: subjects with a definite diagnosis of a syndrome identified in *Step1#*, with no previous orthodontic treatment, reporting no previous conditions associated with dentofacial trauma, and exhibiting the SCIII phenotype; *(I) intervention*: clinical and radiological diagnosis of SCIII and a definite diagnosis of the specific syndrome; *(C) comparison*: group of subjects with no previous orthodontic treatment and non-affected by any syndromic condition neither affected by dentofacial trauma. These subjects could be included in samples belonging to historical datasets from specific growth or developmental studies; *(O) outcomes:* (1) *primary outcome:* to define the phenotypic characteristics of syndromic patients with a SCIII phenotype; (2) *secondary outcome*: to establish the relevant genotype-phenotype associations; *(S) studies*: randomized clinical trials, case-control studies, cross-sectional and cohort studies describing cephalometric studies of syndromic subjects versus controls.

Animal studies, clinical case reports, case series, pilot studies, reviews, books, and book chapters were excluded.

### Data collection and extraction process

Two researchers working independently (M.C.F.T. and C.T.) performed the literature search and compiled the pre-piloted dataset required. Titles, abstracts, and full texts were screened sequentially for eligibility criteria. Discrepancies were resolved by consensus or by a third reviewer (A.I.L). The selection process was as follows: (i) removal of duplicates; (ii) assessment of titles and abstracts to exclude non-relevant studies; (iii) full-text reviews of each study and recording of reasons for exclusion (Supplementary File 3). The *kappa index* was performed to assess inter-rater agreement.

A pre-piloted extraction form was used for data extraction. The data extracted for this scoping review included: key information to identify the study [author, year of publication, type of study], sample characteristics [ethnicity, population, age, sample size, sex, type of syndrome], phenotyping aspects [type (2D or 3D) of cephalometric analysis and related variables, and craniofacial phenotype] (Supplementary File 4) and syndrome reference (OMIM/MalaCards) for each syndrome. Scoping review findings are summarized in Tables 1 and 2. When additional information was needed the corresponding author of each paper would be contacted directly.

**Table 1:** Studies characteristics [Search strategy #Step 2 results].

Authors and year of Publication	Type of Study	Sample characteristics						Analysis	Cephalometric variables							Craniofacial phenotype	Syndrome reference
		Ethnicity	Population	Age	Sample Size	Gender (♀/♂)	Syndrome		Sagital component				Vertical component		Dentoalveolar variables		
Kreiborg S et al. (1999)^56^	Case Control study	-	Denmark	Syndromic group: Age range: 13–35 y Control group: Age range: 13–30 y	Syndromic group: *n* = 26 Control group: *n* = 153	Syndromic group: 15♂ / 11♀ Control group: 51♀ / 102♂	Apert S.	2D	Maxilla: Sp-Pm Cases: 47.8 mm (±3.6) Controls: 58.7 mm(±2.7) s-n-ss Cases: 68.8 mm (±5.4) Controls: 82.0 mm(±3.2) s-n-sp Cases: 76.2 mm (±5.4) Controls: 87.6 mm(±3.3) Mandible: Pgn-Cd Cases: 115.9 mm (±6.8) Controls: 126.1 mm(±5.1) s-n-pg Cases: 75.8º (±7.2) Controls: 81.2º(±3.1) Cranial base: n-ba Cases: 101.9 mm (±6.8) Controls: 112.2 mm (±4.3) s-ba Cases: 49.2 mm (±2.9) Controls: 107.8 mm (±5.7) n-s-ba Cases: 131.8º (±12.1) Controls: 130.2 mm (±5.60) IMM relationship ss-n-pg Cases: −7.0º (±6.1) Controls: 0.4º (±3.1)				NL/ML Cases: 31.1º(±11.1) Controls: 21.0º (±5.9) Facial heights: n-sp Cases: 46.6mm(±6.2) Controls: 56.0mm (±3.0) n-gn Cases: 133.7mm(±10.7) Controls: 126.6mm (±6.5)		-	Class III with midface hypoplasia	OMIM #101200
Lu X et al. (2019)^49^	Case Control study	-	China Brasil USA	Syndromic group: - Control group: -	Syndromic group: *n* = 31 Control group: *n* = 51	-	Apert S.	2D	Apert S. type I Maxilla: SNA Cases: 77.90º (±9.21) Controls: 85.98º (±3.86) ANS-PNS Cases: 24.52mm (±6.60) Controls: 36.18 mm (±5.46) Mandible: N-S-GN Cases: 66.79º (±11.43) Controls: 58.63º (±4.92) COR-COL Cases: 56.48mm (±11.4) Controls: 67.63º (±9.28) GOR-GOL Cases: 51.20mm (±11.54) Controls: 62.47º (±9.02) Cranial base: N-S Cases: 43.16 mm (±7.81) Controls: 49.37 mm(±7.54) IMM relationship ANB Cases: 0.11º (±8.74) Controls: 5.49º (±3.68)	Apert S. type II Maxilla: SNA Cases: 67.00º (±4.24) Controls: 84.86º (±5.41) Mandible: SNB Cases: 72.98º (±4.23) Controls: 80.98º (±7.30) N-S-GN Cases: 78.21º (±2.05) Controls: 64.35º (±5.25) S-N-POG Cases: 71.65º (±4.84) Controls: 80.92º (±7.28) IMM relationship Wits Cases: −8.60º (±5.72) Controls: 3.24º (±3.31)			Apert S. type I SN/Mx Cases: 14.71º (±5.89) Controls: 5.61º (±4.28) Facial heights: S-Pog Cases: 59.09 mm(±14.95) Controls: 73.99 mm(±15.24) S-GN Cases: 59.75mm(±15.73) Controls: 75.60 mm(±15.69) S-GO Cases: 47.10mm(±12.90) Controls: 55.18 mm(±10.88)	Apert S. type II SN/Mx Cases: 14.81º (±7.06) Controls: 6.75º (±3.1) SN/MP Cases: 53.66º (±3.77) Controls: 34.34º (±4.93)	-	Class III with midface hypoplasia	OMIM #101200
Lu X et al. (2019)^50^	Case Control study	-	USA	Syndromic group: 3 subgroups: 0–6 m 6 m-2 y 2years-6 y (24 y) Control group: 3 subgroups: 0–6 m 6 m–2 y 2–6 y (24 y)	Syndromic group: *n* = 18 Control group: *n* = 36	-	Apert S.	2D and 3D	Maxilla: SNA Cases: 73.88º (±8.24) Controls: 86.11º (±3.88) N-S-PP Cases: 76.22º (±7.86) Controls: 75.69º (±3.81) Mandible: SNB Cases: 75.51º (±5.74) Controls: 80.70º (±4.68) N-S-GN Cases: 72.25º (±8.73) Controls: 59.35º (±4.92) S-N-Pog Cases: 73.61º (±5.46) Controls: 80.43º (±4.76) N-S-AR Cases: 104.54º (±5.31) Controls: 104.85º (±3.03) IMM relation: ANB Cases: -1.67º (±7.07) Controls: 5.05º (±3.61) Cranial base: S-N Cases: 48.37mm (±8.23) Controls: 51.77 mm (±9.74) S-ANS Cases: 51.20 mm (±8.39) Controls: 61.16mm (±12.51) S-PNS Cases: 26.21 mm (±3.82) Controls: 32.04mm (±8.57) S-AR Cases: 37.37mm (±6.72) Controls: 42.41mm (±6.13)				SN/Mx Cases:13.79 º (±4.78) Controls: 5.40º (±4.02) FH/Mx Cases:3.97º (±6.01) Controls: 0.28º (±4.28) SN/MP Cases:39.47º (±10.28) Controls: 30.47º(±6.17) FH/MP Cases: 29.65º (±9.15) Controls: 24.98º (±5.62) SN/FH Cases:9.82º (±4.25) Controls: 5.12º (±2.84) Facial heights: S-Pog Cases: 73.29mm (±22.87) Controls: 80.88mm (±21.85) S-GN Cases: 74.04mm (±23.80) Controls: 82.26mm (±21.13) S-GO Cases: 46.37mm (±13.90) Controls: 49.49mm (±13.61) ANS-N Cases: 29.60mm (±6.10) Controls: 31.51mm (±8.16) ANS-Me Cases: 46.36mm (±20.70) Controls: 43.78mm (±13.89) N-Me Cases: 75.00mm (±26.75) Controls: 75.20mm (±21.64)		-	Class III with midface hypoplasia	OMIM #101200
Reitsma JH et al. (2019)^51^	Case Control study	-	The Netherlands	Syndromic group: Age range: 8.2–19.8 y Control group: Age range: 4–15 y	Syndromic group: *n* = 13 Control group: *n* = 486	-	Apert S. Crouzon S.	2D	Apert S. Group: Maxilla: SNA Cases: 64.69º (±5.48) Controls: 80.56 º (±3.69) Mandible: SNB Cases: 78.35º (±4.79) Controls: 77.39 º (±3.44) IMM relationship ANB Cases: -13.56º (±4.94) Controls: Controls: 3.73 º (±2.48)	Crouzon S. Group: Maxilla: SNA Cases: 70.14º (±2.61) Controls: 80.39 º (±3.81) Mandible: SNB Cases: 80.28º (±2.71) Controls: 77.08 º (±3.37) IMM relationship ANB Cases: -10.21º (±3.04) Controls: 3.96 º (±2.35)			Apert S. Group: SN/PP Cases: -11.39º (±8.46) Controls: 8.50 º (±2.77) Facial heights: LFH ratio Cases: 74.78% (±1.93) Controls: 56.83 º (±2.45)	Crouzon S. Group: SN/PP Cases: -0.15º (±7.78) Controls: 8.31 º (±3.24) Facial heights: LFH ratio Cases: 64.69% (±5.76) Controls: 57.15 º (±2.45)	-	Class III with midface hypoplasia	OMIM #101200 OMIM #123500
Engel M et al. (2019)^55^	Case Control study	-	Germany	Syndromic group: Median age: 12.5 y Control group: -	Syndromic group: *n* = 9 Control group: *n*= -	Syndromic group: 6♂ / 3♀ Control group: -	Crouzon S.	2D	Maxilla SNA Cases: 76.0º(±2.90) Controls: 81.0º (±3.0) IMM relationship ANB Cases: -4.8º (±3.7) Controls: 2º(±2)				-		-	Class III with midface hypoplasia	OMIM #123500
Lu X et al. (2020)^52^	Case Control study	Asian Brazilian Caucasian	China Brasil USA	Syndromic group: Mean age: Caucasian Crouzon:9.51y Asian Crouzon: 9.71y Control group: Mean age: Caucasian patients: 9.87y Asian patients:9.90y	Syndromic group: *n* = 28 Control group: *n* = 63	-	Crouzon S.	2D and 3D	Maxilla: SNA Asian Cases: 70.56º (±6.21) Controls: 80.31º (±3.23) Caucasian Cases: 75.65º (±7.15) Controls: 84.08º (±4.57) ANS-PNS Asian Cases: 38.37mm (±4.26) Controls: 44.73mm (±7.24) Caucasian Cases: 41.95mm (±7.28) Controls: 46.83mm (±8.50) Mandible: N-A-Pog Asian Cases: 195.54º (±8.32) Controls: 170.77º (±5.42) Caucasian Cases: 180.41º (±12.27) Controls: 180.95º (±10.81) S-N-Pog Asian Cases: 78.37º (±8.52) Controls: 75.98º (±3.72) Caucasian Cases: 75.35º (±6.02) Controls: 79.23º (±5.49) CO-GO/GO-Pog Asian Cases: 0.70º (±0.07) Controls: 0.64º (±0.05) Caucasian Cases: 0.67º (±0.06) Controls: 0.64º (±0.06) IMM relationship ANB Asian Cases: −8.33º (±3.69) Controls: 4.84º (±1.94) Caucasian Cases: −2.65º (±5.22) Controls: 4.37º (±2.19) Wits Asian Cases: −9.92mm (±3.49) Controls: 0.25 mm (±3.52) Caucasian Cases: −4.38mm (±5.84) Controls: 1.14mm (±3.71) Cranial base: S-A Asian Cases: 1.21mm (±0.14) Controls: 1.14mm(±0.08) Caucasian Cases:1.23mm (±0.19) Controls: 1.04mm (±0.13) S-ANS Asian Cases: 61.45mm (±7.48) Controls:72.11mm(±10.35) Caucasian Cases: 62.43mm (±9.10) Controls: 74.36mm (±10.63) S-PNS Asian Cases:35.63mm (±6.86) Controls: 41.47mm(±6.81) Caucasian Cases: 31.40mm (±7.11) Controls: 40.41mm (±6.75) S-Ar Asian Cases: 46.91mm (±7.07) Controls: 53.80mm (±6.72) Caucasian Cases: 41.87mm (±4.89) Controls: 48.20mm (±9.02)				SN/Mx Asian Cases:9.89º (±5.04) Controls: 8.69º(±3.09) Caucasian Cases: 11.89º (±4.70) Controls: 6.35º (±3.90) SN/MP Asian Cases: 39.15º (±11.61) Controls: 38.69º(±5.49)Caucasian Cases: 44.93º (±8.51) Controls: 33.89º (±5.56)		-	Class III with midface hypoplasia	OMIM #123500
Cardoso R et al. (2012)^15^	Case control study	-	Brazil	Syndromic group: Age range: 18–53 y Control group: -	Syndromic group: *n* = 14 Control group: -	Syndromic group: 6♂ / 8♀ Control group: -	Achondroplasia	2D	Maxilla SNA Cases: 82.31º (±3.16) Controls: 82º (−) Maxillary depth Cases: 88.23 mm (±4.93) Controls: 90.0 mm (±3.0) Mandible SNB Cases: 83.22º (±5.76) Controls: 80.0º (-) SND Cases: 80.1º (±6.0) Controls: 76º (-) Mandibular body lenght Cases: 73.41 mm (±7.67) Controls: 81.0 mm (±2.5) IMM relationship ANB Cases: 0.11º (±4.53) Controls: 2º (-) Cranial base S-N Cases: 70.64mm (±2.62) Controls: 78.0mm (±3.0) S-Ar Cases: 27.0mm (±4.04) Controls: 36.5mm (±3.0) N-S-Ar Cases: 117.29º (±10.0) Controls: 130.0º (±5.0)				Palatal plane: Cases: 5.8º (±5.1) Controls: 1º (±3.5) Mandibular plane: Cases: 29.1º (±5.5) Controls: 24.0º (±4.0) Maxillary height Cases: 55.8mm (±2.4) Controls: 57.0mm (±3.0) Posterior facial height Cases: 64.7mm (±3.7) Controls: 65.0mm (±3.3) Total facial height: Cases: 63.1mm (±3.68) Controls: 60.0mm (±3.0)		1/.NA Cases: 40.74º (±27.21) Controls: 22º 1/-NA Cases: 11.36mm (±4.82) Controls: 4mm 1/.NB Cases: 27.70º (±8.14) Controls: 25º 1/-NB Cases: 7.52mm (±2.70) Controls: 4mm	Class III with midface hypoplasia and mandibular prognathism	OMIM #100800
Arslan S G et al. (2007)^20^	Case Control study	-	Turkey	Syndromic group: Age range: 6.5–17 y Control group: Age range: 7–18 y	Syndromic group: *n* = 10 Control group: *n* = 10	Syndromic group: 6♂ / 4♀ Control group: 6♂ / 4♀	X Linked Hypohidrotic Ectoderrmal dysplasia	2D	Maxilla SNA Cases: 76.4º(±3.58) Controls: 77.6º(±1.77) Mandible SNB Cases: 76.9(±4.36) Controls: 77.7º(±2.35) IMM relationship ANB Cases: -0.5º (±5.68) Controls: −0.1º(±1.37)				SN/GoMe Cases: 27.9º(±5.71) Controls: 28.85º(±2.94) Jarabak ratio SGo/NMe% Cases: 66.88º(±4.26) Controls: 70.98º(±3.39)		-	Class III with maxillary hypoplasia and mandibular prognathism	OMIM #305100
Sonnesen L et al. (2018)^54^	Case-control study	-	Denmark	Syndromic group: Age range: 8–16 y Control group: Age range: 9–16 y	Syndromic group: *n* = 15 Control group: *n* = 22	Syndromic group: 12♂ / 3♀ Control group: 8♂ / 14♀	X Linked Hypohidrotic Ectoderrmal dysplasia	2D	Maxilla s-n-ss Cases: 122.2º (±4.9) Controls: 125.3 º (±4.1) Mandible s-n-pg Cases: 81.5º (±5.0) Controls: 78.1 º (±4.0) IMM relationship ss-n-pg Cases: -3.3º (±4.0) Controls: 1.7º (±1.3) Cranial base n-s-ba Cases: 129.6º (±3.9) Controls: 131.4º (±4.8) n-s-ar Cases: 122.2º (±4.9) Controls: 125.3º (±4.1)				NSL/NL Cases: 4.1º (±3.7) Controls: 8.5º(±3.7) NSL/ML Cases: 26.7º (±5.8) Controls: 32.4º(±4.3) NL/ML Cases: 22.6º (±5.0) Controls: 23.9º (±2.9)		-	Class III with maxillary hypoplasia and mandibular prognathism	OMIM #305100
Lexner et al. (2007)^53^	Case Control study	-	Denmark	Syndromic group: Age range: ♂ 19–49 y Age range : ♀ 17–75 y Control group: Age range : 5–31 y	Syndromic group: *n* = 43 Control group: *n* = 469	Syndromic group: 10♂ / 33♀ Control group: 205♂ / 264♀	X Linked Hypohidrotic Ectoderrmal dysplasia	2D	Maxilla sp-pm Cases: ♂49.5mm (±3.1) ♀50.3mm (±3.0) Controls: ♂55.6mm (±2.5) ♀52.6mm (±2.6) s-n-ss Cases: ♂78.9º (±2.7) ♀78.8º (±4.1) Controls: ♂82.0º (±3.2) ♀81.5º (±3.5) Mandible ar-go Cases: ♂51.5mm (±5.9) ♀46.3mm (±4.9) Controls: ♂50.7mm (±4.6) ♀46.5mm (±3.7) s-n-pg Cases: ♂87.9º (±6.1) ♀80.7º (±4.9) Controls: ♂81.2º (±3.1) ♀80.5º (±3.2) IMM relationship ss-n-pg Cases: ♂-9.0º (±6.9) ♀-2.0º (±4.4) Controls: ♂0.4º (±3.1) ♀1.0º (±1.9) Cranial base n-s Cases: ♂71mm (±3.4) ♀68mm (±3.5) Controls: ♂70.1mm(±3.0) ♀67.1mm (±2.3) n-ba Cases: ♂105.5mm (±3.5) ♀101.5mm (±5.1) Controls: ♂106.2mm(±4.1) ♀100.4mm (±3.2) n-s-ba Cases: ♂128.8º (±5.2) ♀132.1º (±3.9) Controls: ♂130.2º (±5.6) ♀ 129.3º (±4.9)				ML/RL Cases: ♂ 120.3º (±9.9) ♀ 119.7º (±7.4) Controls: ♂ 120.4º (±6.3) ♀ 121.2º (±5.2) NSL/ML Cases: ♂ 20.7º (±7.2) ♀ 27.9º (±7.7) Controls: ♂ 28.2º (±5.9) ♀ 29.6º (±5.7) NL/ML Cases: ♂ 18.2º (±5.7) ♀ 20.5º (±6.5) Controls: ♂ 21.0º (±5.9) ♀ 22.0º (±5.1)		-	Class III with maxillary hypoplasia and mandibular prognathism	OMIM #305100
Johnson EL et al. 2002^48^	Case-Control Study	-	USA	Syndromic group: 4 subgroups: 0–5 y 5–12 y 13–17 y >17 y Control group: 4 sub groups: 0–5 y 5–12 y 13–17 y >17 y	Syndromic group: *n* = 95 Control group: *n* = 128	Syndromic group: 95 ♂ Control group: 128 ♂	X Linked Hypohidrotic Ectoderrmal dysplasia	2D	ED 0-5 years group Maxilla SNA Cases: 83.2º (±2.0) Controls: 83.5º(±3.9) ANS-PNS Cases:37.7mm (±6.6) Controls: 39.3mm(±3.6) Mandible Go-Ar Cases:36.3 mm(±1.0) Controls: 34.3 mm(±1.9) Go-Pg Cases:53.7 mm(±3.4) Controls: 58.1 mm(±3.1) Pg-N-S Cases: 81.4º (±2.0) Controls: 77.8º (±2.8) Cranial base S-N Cases: 61.1 mm (±2.3) Controls: 62.3mm(±2.9) Ba-N Cases:85.2 mm (±2.8) Controls: 85.6mm(±2.9) S-Ba Cases: 35.5 mm (±2.8) Controls: 125.3mm(±4.1) S-ANS Cases:69.1mm (±4.1) Controls: 72.0mm(±3.2) Ba-S-N Cases: 121.8º(±3.1) Controls: 128.9º (±3.6)	ED 5-12 years group Maxilla SNA Cases: 79.0º (±4.5) Controls: 83.9º(±3.8) ANS-PNS Cases:42.1mm (±2.3) Controls: 44.8mm(±3.3) Mandible Go-Ar Cases:40.8 mm(±7.0) Controls: 39.4 mm(±3.8) Go-Pg Cases:70.8 mm(±4.8) Controls: 68.9 mm(±5.5) Pg-N-S Cases: 84.9º (±5.7) Controls: 79.5º (±3.1) Cranial base S-N Cases: 66.8 mm (±4.9) Controls: 67.5mm(±3.8) Ba-N Cases: 100.6 mm (±4.7) Controls: 95.8mm(±5.8) S-Ba Cases: 45.6 mm (±2.6) Controls: 38.6mm(±3.9) S-ANS Cases:76.9mm (±3.9) Controls: 79.8mm(±3.2) Ba-S-N Cases: 124.5º(±8.0) Controls: 127.1º (±3.9)	ED 13-17 years group Maxilla SNA Cases: 79,1º (±6,2) Controls: 84,2º(±4,0) ANS-PNS Cases:46,6mm (±2,6) Controls: 51,,3mm(±3,4) Mandible Go-Ar Cases:46,4 mm(±4,8) Controls: 48,7 mm(±3,6) Go-Pg Cases:78,9 mm(±5,5) Controls: 80,0 mm(±4,2) Pg-N-S Cases: 85,2º (±5,7) Controls: 81,9º (±3,4) Cranial base S-N Cases: 69,7 mm (±4,1) Controls: 73,1mm(±3,5) Ba-N Cases: 107,0 mm (±5,9) Controls: 106,2mm(±4,3) S-Ba Cases: 51,9 mm (±1,9) Controls: 43,8mm(±4,0) S-ANS Cases:82,2mm (±6,1) Controls: 88,5mm(±4,4) Ba-S-N Cases: 127,7º(±7,8) Controls: 128,9º (±3,6)	ED >17 years group Maxilla SNA Cases: 77.6º (±5.3) Controls: 83.9º(±3.7) ANS-PNS Cases:46.8mm (±5.9) Controls: 52.8mm(±3.1) Mandible Go-Ar Cases:46.1 mm(±5.8) Controls: 52.3 mm(±3.1) Go-Pg Cases:81.2 mm(±5.2) Controls: 82.5 mm(±4.1) Pg-N-S Cases: 84.2º (±4.8) Controls: 82.9º (±3.5) Cranial base S-N Cases: 71.4 mm (±4.3) Controls: 74.8mm(±3.3) Ba-N Cases: 108.1 mm (±9.0) Controls: 108.5mm(±3.8) S-Ba Cases: 50.9 mm (±3.6) Controls: 45.2mm(±3.1) S-ANS Cases:82.6mm (±6.6) Controls: 90.18mm(±3.8) Ba-S-N Cases: 123.5º(±6.0) Controls: 128.1º (±4.6)	ED 0-5 years group NL/ML Cases: 20.4º (±7.4) Controls: 26.0º (±5.2) ArGoMe Cases: 134.2º (±8.7) Controls: 130.7º (±3.5) Facial heights: S-Pg Cases:93.0mm (±0.5) Controls: 95.2mm(±4.5) N-ANS Cases:37.1mm (±0.93) Controls: 39.6mm(±2.0) N-Me Cases:82.2mm (±3.6) Controls: 90.8mm(±2.9) ED 5-12 years group NL/ML Cases: 24.1º (±4.9) Controls: 26.5º (±4.4) ArGoMeCases: 128.3º (±4.8) Controls: 128.8º (±4.8) Facial heights: S-Pg Cases:111.2mm (±7.5) Controls: 109.1mm(±7.0) N-ANS Cases:44.9mm (±3.6) Controls: 46.3mm(±4.3) N-Me Cases:99.7mm (±7.4) Controls: 103.6mm(±6.4)	ED 13-17 years group NL/ML Cases: 27,6º (±5,5) Controls: 24,5º (±5,2) ArGoMe Cases: 127,7º (±4,8) Controls: 126,2º (±4,8) Facial heights: S-Pg Cases:123,9mm (±10,7) Controls: 125,95 mm(±5,9) N-ANS Cases:50,6mm (±4,9) Controls: 53,6mm(±3,5) N-Me Cases:114,3mm (±9,5) Controls: 118,9mm(±5,1) ED >17 years group NL/ML Cases: 30.8º (±3.7) Controls: 23.1º (±5.6) ArGoMe Cases: 130.8º (±2.3) Controls: 124.3º (±4.8) Facial heights: S-Pg Cases:131.5mm (±5.9) Controls: 130.8mm(±5.0) N-ANS Cases:54.6mm (±4.4) Controls: 54.5mm(±3.6) N-Me Cases:123.1mm (±8.5) Controls: 122.4mm(±5.4)	-	Class III with maxillary hypoplasia and mandibular prognathism	OMIM #305100
Nguyen T et al. 2013^21^	Familliar case control study	-	-	Syndromic group: Age range: 5.0–79.3 y Control group: Age range: 5.8–76.0 y	Syndromic group: *n* = 53 Control group: *n* = 34	Syndromic group: 28♂ / 25♀ Control group: 16♂ / 18♀	Tricho-dento-osseous syndrome	2D	Maxilla SNA Cases:78.84º (±0.45) Controls: 81.50 º (±0.53) Co-A Cases:91.47 mm(±0.87) Controls: 91.14 mm (±1.14) Mandible SNB Cases:77.58 º (±0.51) Controls: 78.22º (±0.62) Co-Gn Cases:126.65 mm(±1.25) Controls: 78.22 mm (±0.62) Go-Gn Cases:82.47 mm (±0.95) Controls: 78.87 mm (±1.25) Go-Pg Cases:78.9 mm (±5.5) Controls: 80.0 mm (±4.2) Pg-N-S Cases:85.2º (±5.7) Controls: 81.9º (±3.4) Cranial base S-N Cases:75.96 mm (±0.67) Controls: 75.01 mm (±0.7) S-ANS Cases:82.2 mm (±6.1) Controls: 88.5 mm (±4.4) S-Ba Cases:40.15 mm (±0.78) Controls: 38.51 mm (±0.85) S-Pg Cases:123.9 mm (±10.7) Controls: 125.95 mm (±5.9) Ba-S-N Cases:129.42º (±0.58) Controls: 128.72º (±0.81) IMM relation: ANB Cases: 1.27º (±0.39) Controls: 3.29º (±0.3)				Ar-Go-Me Cases: 128.18º(±0.80) Controls: 128.14º (±1.26) Facial heights N-Me Cases: 122.15mm (±1.71) Controls: 122.88mm (±1.88) N-ANS Cases: 65.7 mm(±5.56) Controls: 72.9mm(±5.56) ANS-Me Cases:69.55 mm (±1.29) Controls: 70.29 mm (±1.37)		-	Class III with maxillary hypoplasia	OMIM #190320
Jensen BL 1994^22^	Case Control study	-	Denmark USA The Netherlands Scotland	Syndromic group: Age range: 16–58 y Control group: Age range: 20–30 y	Syndromic group: *n* = 35 Control group: *n* = 153	Syndromic group: 15♂ / 20♀ Control group: 102♂ / 51♀	Cleidocranial dysplasia	2D	Maxilla s-pm Cases: ♂45.77mm (±3.45) ♀41.81mm (±2.45) Controls: ♂49.57 mm (±2.81) ♀47.41 mm (±2.35) s-n-sp Cases: ♂96.30º (±6.67) ♀97.53º (±5.24) Controls: ♂87.64º (±3.28) ♀87.74º (±4.02) s-n-ss Cases: ♂88.42º (±7.21) ♀91.12(±5.22) Controls: ♂81.95º (±3.19) ♀ 81.53º (±3.50) Mandible pgn-cd Cases: ♂121.19 mm (±7.89) ♀114.67 mm (±4.82) Controls: ♂126.11 mm (±5.05) ♀118.91 mm (±4.91) s-n-pg Cases: ♂90.93 º (±3.62) ♀90.85º (±3.78) Controls: ♂81.23º (±3.14) ♀80.45º (±3.21) IMM relationship ss-n-pg Cases: ♂-2.28º (±4.59) ♀0.17º (±4.74) Controls: ♂0.41º (±3.10) ♀1.02º (±1.85) Cranial base s-n Cases: ♂70.38 mm(±3.65) ♀64.42mm (±4.09) Controls: ♂74.0mm (±3.13) ♀70.90mm (±2.39) s-ba Cases: ♂44.1 mm(±4.51) ♀42.77mm(±3.77) Controls:♂49.20º (±2.93) ♀45.87mm (±2.27) Cranial base angle n-s-ba Cases: ♂122.90º (±7.16) ♀128.92º (±6.18) Controls: ♂130.18º (±5.55) ♀129.28º (±4.89)				NSL/NL Cases: ♂6.41º (±3.76) ♀5.30º (±3.14) Controls: ♂7.66º (±3.18) ♀7.13º (±3.19) NSL/ML Cases: ♂17.96º (±6.53) ♀21.44º (±6.95) Controls: ♂28.22º(±5.85) ♀29.64º(±5.72) NL/ML Cases: ♂11.41º (±7.82) ♀16.14º (±6.75) Controls: ♂21.04º (±5.92) ♀21.95º (±5.08) ML/MR Cases: ♂117.27º (±7.46) ♀119.98º (±8.61) Controls: ♂120.38º (±6.34) ♀121.17º (±5.20) Facial heights: n-sp Cases: ♂49.32 mm(±4.68) ♀45.44 mm (±2.92) Controls: ♂56.02mm(±3.02) ♀52.82mm (±2.74) n-gn Cases: ♂113.11 mm(±7.94) ♀106.90 mm (±7.13) Controls: ♂126.60mm(±6.48) ♀119.64mm (±6.35)		-	Class III with mandibular prognathism	OMIM #119600
Brkic H et al. 1994^47^	Case Control study	-	Croatia	Syndromic group: Mean age: 27 y Control group Age range: 21–27 y	Syndromic group: *n* = 35 Control group: *n* = 60	Syndromic group: 35 ♂ Control group: 60 ♂	Klinefelter S.	2D	Maxilla: sp-pm Cases: 57.98 mm(±3.64) Controls: 59.98mm(±5.45) s-n-sp Cases: 90.85 º(±4.52) Controls: 88.48 º(±4.01) Mandible: pgn-cd Cases: 131.74 mm(±4.11) Controls: 129.41mm(±4.09) s-n-pg Cases: 85.34 (±3.66) Controls: 82.58mm(±3.75) Cranial base: n-s Cases: 75.07mm(±4.14) Controls: 77.07mm(±3.21) s-ba Cases: 49.36mm (±4.25) Controls: 51.39mm(±3.99) n-s-ba Cases: 124.8º (±4.47) Controls: 127.45mm(±5.90)				NSL-NL Cases: 8.26º (±3.03) Controls: 7.23mm(±3.54) NSL/ML Cases: 31.08º (±6.75) Controls: 27.31º(±6.99) NL/ML Cases: 24.54º (±7.63) Controls: 23.26º (±6.72) ML/MR Cases: 127.70º (±6.32) Controls: 120.86º (±10.36) Facial heights: n-sp Cases: 56.12mm(±5.37) Controls: 57.52mm(±4.16) sp-gn Cases: 74.54mm (±6.94) Controls: 75.06mm(±6.23)		ILs/NL Cases: 110.03º (±7.78) Controls: 108.63º (±6.54) ILI/ML Cases: 85.11º (±8.48) Controls: 93.08º (±6.58) ILs/Ili Cases: 137.64º (±9.31) Controls: 134.71º (±8.92)	Class III with mandibular prognathism	MCID: HYP730
Ingerslev C H et al. 1978^32^	Case Control study	-	Denmark	Syndromic group: adults Control group: adults	Syndromic group: *n* = 37 Control group: *n* = 102	Syndromic group: 37 ♂ Control group: 102 ♂	Klinefelter S.	2D	Maxilla: sp-pm Cases: 57.64 mm(±3.82) Controls: 58.16mm (±2.83) Mandible: pgn-cd Cases: 126.05 mm(±5.76) Controls: 125.85mm(±4.97) s-n-pg Cases: 84.64 º(±0,61) Controls: 80,97º (±0,13) IMM relationship: ss-n-pg Cases: 1.91º (±0.41) Controls: 0.23º mm (±0.29) Cranial base: n-s Cases: 71.58mm(±2.70) Controls: 73.38 mm (±3.11) s-ba Cases: 47.62mm (±3.20) Controls: 48.93mm (±2.86) n-s-ba Cases: 128.2º (±5.65) Controls: 131.51mm (±5.39) n-s-ar Cases: 119.48º (±5.90) Controls: 123.23º (±5.69)				NSL/NL Cases: 5.00º (±3.67) Controls: 7.62mm(±2.95) NSL/ML Cases: 29.52º (±5.09) Controls: 28.00º(±5.89) NL/ML Cases: 24.76 (±5.06) Controls: 20.34º (±5.75) ML/MR Cases:125.08º (±6.39) Controls: 120.14 º(±6.33) Facial heights: n-sp Cases: 53.50 mm(±3.60) Controls: 55.66mm(±3.07) sp-gn Cases: 71.86 mm (±5.19) Controls: 72.24mm(±5.24)		ILs/NL Cases: 111.55º (±8.68) Controls: 110.85º (±6.51) ILI/ML Cases: 92.24º (±6.94) Controls: 98.47º (±7.26) ILs/Ili Cases: 130.09º (±13.74) Controls: 131.73º (±10.98)	Class III with mandibular prognathism	MCID: HYP730
Brown T et al.1 993^33^	Familliar case control study	-	Finland	Syndromic group: Age range: 5–58 y Control group : Age range: ♂: 18–67 y ♀: 26–51 y	Syndromic group: *n* = 40 Control group: *n* = 33	Syndromic group: 40 ♂ Control group: 17♂ / 16♀	Klinefelter S.	2D	Maxilla ss-pm Cases: 50.18mm(±2.73) Controls: 48.70 mm (±3.60) s-n-sp Cases: 90.90 º(±4.77) Controls: 87.98 º(±5.02) Mandible mandibular corpus lenght (MC) Cases: 76,56mm(±5.7) Controls: 71,30mm (±6.65) s-n-pg Cases: 84.45º(±5.12) Controls: 81.88 mm (±2.91) pg-tgo Cases: 81.36mm (±5.96) Controls: 76.19 mm (±7.30) s-n-sm Cases: 85.10º(±5.06) Controls: 80.45 mm (±2.91) IMM relationship: ss-n-sm Cases: 1.23º (±3.45) Controls: 2.36º mm (±3.56) Cranial base n-ba Cases: 105.65mm (±4.28) Controls: 104.33 mm (±4.07) s-ar Cases: 35.03 mm(±3.59) Controls: 37.41 mm (±2.60) n-s-ba Cases: 127.73º (±6.22) Controls: 126.78 mm (±4.55) n-s-ar Cases: 120.35º (±5.68) Controls: 122.03 mm (±4.39)				ML/NSL Cases: 28.47º(±7.05) Controls: 27.85º (±5.09) ML/MR Cases: 123.22º (±6.99) Controls: 121.51º (±8.18) Ar-Tgo Cases: 51.06 mm(±4.16) Controls: 53.99 mm (±4.20) Facial heights: n-gn Cases: 118.62mm(±6.78) Controls: 120.81 mm (±7.31) sp-gn Cases: 68.38mm (±5.77) Controls: 70.21 mm (±5.67)		-	Class III with mandibular prognathism	MCID: HYP730
Babić M et al. 1993^31^	Case Control study	-	Yugoslavia	Syndromic group: Age range: 22–31 y Control group: Age range: 22–26 y	Syndromic group: *n* = 43 Control group: *n* = 93	Syndromic group: 28♂ / 15♀ Control group: 31♂ / 62 ♀	Klinefelter S.	2D	Effects of X-Chromosome aneuploidy in Klinefelter Syndrome Maxilla SNA Cases:85.2º(±3.7) Controls: 81.9 º (±3.7) ANS-PNS Cases: 55.0mm (±3.9) Controls:60.3 mm(±4.0) Mandible SNB Cases:85.5(±4.4) Controls: 79.2 º (±3.5) Go-Pg Cases :85.7mm (±3.9) Controls: 85.2 mm(±5.9) Go-Cd Cases :67.5 mm(±4.7) Controls: 72.5 mm(±3.3) IMM relationship ANB Cases: -0.3º (±3.1) Controls: 2.8 º(±2.4) Cranial base N-S Cases: 74.5mm(±3.2) Controls: 77.3 mm(±3.5) S-Ba Cases: 50.8º(±3.3) Controls: 51.7 mm(±3.4) NSBa Cases: 123.3º (±6.0) Controls: 128.7 º(±4.9)				Effects of X-Chromosome aneuploidy in Klinefelter Syndrome NS/SpP Cases: 80.0º(±3.7) Controls: 3.4º (±4.7) NS/MP Cases:25.5 (±5.7) Controls: 28.2 º (±5.3) SpP/MP Cases: 22.3º(±6.4) Controls: 20.4º(±5.5) ArGoMe Cases: 122.5º (±5.7) Controls: 119.4 º(±6.5) Facial heights N-MeCases: 125.6mm(±6.4) Controls: 133.7 mm(±6.0) S-Go Cases: 91.3mm (±5.6) Controls: 95.6 mm(±5.2) S-Go/N-Me (%) Cases:72.8(±4.8) Controls: 71.6 mm(±4.2)		-	Class III with mandibular prognathism	MCID: HYP730
Suri S et al. 2010^27^	Case Control study	-	Canada	Syndromic group: Age range: 11.5–18.3 y Mean age: 15.1 y Control group: Mean age: 15.1 y	Syndromic group: *n* = 25 Control group: *n* = 25	Syndromic group: 13 ♀ / 12 ♂ Control group: 13 ♀ / 12 ♂	Down S.	2D	Maxilla SNA Cases: 82.47º (±4.34) Controls: 81.25º (±2.87) ANS-PNS Cases: 47.80 mm (±3.77) Controls: 57.90mm (±3.76) Mandible SNB Cases: 82.41º(±4.36) Controls: 78.74º (±2.64) Co-Gn Cases: 112.91 mm(±8.07) Controls: 121.26 (±5.58) Go-Gn Cases: 75.21 mm(±6.60) Controls: 79.72mm (±4.74) IMM relation ANB Cases: 0.06 (±2.51) Controls: 2.52º (±1.48) Cranial base S-N Cases:64.97 mm (±3.52) Controls: 75.17 mm (±3.74) Ba-S Cases: 44.46 mm (±3.05) Controls: 48.40mm (±3.01) Ba-S-Na Cases: 140.31º (±3.75) Controls: 129.92º (±4.06)				S-N/Mx Cases: 8.53 (±2.37) Controls: 8.22 (±2.96) Ba-N/Mx Cases: 28.61º(±6.31) Controls: 27.47º (±2.66) S-N/Go-Gn Cases: 28.61º(±6.31) Controls: 30.34º (±4.50) Co-Go-Gn Cases: 121.68º (±6.50) Controls: 123.12 (±4.95) Facial heights S-Go Cases:70.36 (±5.88) Controls: 78.83 (±6.45) ANS-Me Cases: 61.84 (±6.28) Controls: 69.54 (±5.04) N-ANS Cases:47.42 (±2.89) Controls: 54.65 (±3.01) N-Me Cases: 106.23 (±8.04) Controls: 121.74 (±6.00) S-Go/N-Me (%) Cases: 65.10 (±5.17) Controls: 64.43 (±4.10)		Overjet Cases:-0.26mm (±2.96) Controls: 2.52 mm (±1.09) Overbite Cases: 0.25 (±2.53) Controls: 4.08 (±1.70) U1-Mx Cases: 24.04mm (±3.14) Controls: 30.33 (±2.38) U1- Mx Cases: 63.21º (±6.84) Controls: 70.09º (±5.49) L1-MP Cases: 39.18mm (±3.46) Controls: 42.99mm (±3.35) L1-MP Cases: 92.85º (±7.63) Controls: 93.18º (±5.72) Interincisal angle Cases:126.50º Controls: 131.39 (±7.15)	Class III with midface hypoplasia	OMIM #190685
Korayem MA et al 2014.^30^	Case-Control Study	-	Saudi Arabia	Syndromic group: Age range: 12–22 y Mean age: 15.8 y Control group: Age range: 0–14 y	Syndromic group: *n* = 60 Control group: *n* = 60	Syndromic group: 33♀ / 27♂ Control group: 33♀ / 27♂	Down S.	2D	Maxilla SNA Cases: 81.9º (±2.4) Controls: 83.3º(±2.5) Co-A Cases: 85.7 mm (±5.8) Controls: 92.7mm (±3.0) Mandible SNB Cases: 81.4º(±3.0) Controls: 80.4º (±2.7) Co-Gn Cases: 122.9mm (±5.1) Controls: 124.7mm (±5.4) IMM relation ANB Cases: 0.54º (±2.6) Controls: 3.1º (±0.9) Wits Cases: -1.75mm (±2.9) Controls: -0.47mm (±1.3) Cranial base SN Cases: 65.2mm (±4.4) Controls: 72.9mm (±3.6) SBa Cases: 44.5mm (±3.3) Controls: 46.5mm (±3.3) NSBa Cases: 138.53º (±3.86) Controls: 130.23º (±1.96)				SN-MP Cases: 36.0º (±5.1) Controls:33.7º(±4.4) PP-MP Cases:29.7º (±5.0) Controls:27.9º(±4.2) Ar-Go-Me Cases: 134.1º (±6.9) Controls:127.9º(±3.4) ANS-Me/N-Me Cases: 58.0º (±2.6) Controls:56.6º(±2.3) Y axis (SGn-SN) Cases:71.2º (±4.6) Controls:68.3º(±3.0)		U1-L1 Cases: 116º (±10.7) Controls:127.0º(±5.5) U1-NA Cases:25.9º (±6.9) Controls:23.0º(±3.9) U1-NA Cases: 6.01 mm (±2.8) Controls:4.4mm(±1.8) L1-NB Cases:35.1º (±7.0) Controls:25.8º(±3.2) L1-NB Cases:7.3mm (±2.7) Controls:5.1mm(±1.7)	Class III with midface hypoplasia	OMIM #190685
Silva Jesuino FA et al. 2013^26^	Case-Control Study	Caucasian	Brazil	Syndromic group: Mean Age: 8 y 3 m Control group: 1.Mean Age: 7y 9 m 2. Mean Age: 8 y 2 m	Syndromic group: *n* = 30 Control groups: *n* = 30+30	-	Down S.	2D	Maxilla SNA Cases: 79.9º (±3.91) Controls: 80.9ª(±3.19) Co-A Cases: 75.4mm (±4.56) Controls: 84.8mm(±4.36) Mandible SNB Cases: 78.4º(±4.3) Controls: 77.2º(±3.14) GoGn Cases:96mm(±7.71) Controls: 104.7 mm(±5.48) IMM relation ANB Cases: 1.4º (±2.90) Controls: 3.6º (±1.98) Cranial base S-N Cases: 62.2 mm (±3.78) Controls: 69.6mm(±4.22) Ba-S Cases: 42.2 mm (±3.08) Controls: 44.2mm(±2.84) Ba-N Cases: 96.6 mm (±5.28) Controls: 103.3mm(±5.86) BaSNCases: 134.6º(±4.90) Controls: 128.1mm(±2.85)				ArGoMe Cases: 127.6º (±5.86) Controls: 130.4º (±5.36) Facial heights S-Go Cases: 62.7 mm(±5.9) Controls: 66.7mm(±4.71) Ar-Go Cases: 38.2 mm(±5.24) Controls: 38.2mm(±5.24) ANS-Me Cases: 57mm (±4.50) Controls: 62.1mm(±3.38) N-ANS Cases: 42.2mm (±4.60) Controls: 47.9mm(±5.59) N-Me Cases: 97.7mm (±7.28) Controls: 108.3mm(±5.59)		-	Class III with midface hypoplasia	OMIM #190685
Clarkson C et al. 2004^29^	Case-Control Study	-	Colombia	Syndromic group: Age range 8–11 y Control group : 8–11 y	Syndromic group: *n* = 14 Control group: *n* = 14	-	Down S.	2D	Maxilla SNA Cases: 80.5º (±2.68) Controls: 82.5ª(±2.68) Mandible SNB Cases: 77.2º(±2.5) Controls: 77.8º(±2.5) Wits Cases: -2.1mm(±1.0) Controls: -1mm(±1.0) IMM relationship ANB Cases: 3.3º (±2.48) Controls: 4.7º (±2.48) Cranial base S-N Cases: 63.3 mm (±3.48) Controls: 69.10mm(±3.48)				Facial heights N-Me Cases: 103.5mm (±6.99) Controls: 113.2mm(±6.99) S-Go Cases: 65.7 mm(±5.56) Controls: 72.9mm(±5.56)		-	Class III with midface hypoplasia	OMIM #190685
Fischer-Brandies H 1998^28^	Case-Control Study	-	Germany	Syndromic group: Age range: 0–14 y Control group: Age range: 0–14 y	Syndromic group: *n* = 1896 Control group: *n* = 1154	Syndromic group: 834♀ / 1062 ♂ Control group: 499♀ / 655 ♂	Down S.	2D	Down Syndrome 0-3 months age group Maxilla: SNA Cases: ♂83.6º (±4.7) ♀83.9º (±3.6) Controls: - SpP Cases: ♂24.3 mm (±1.7) ♀24.0mm (±3.1) Controls: - Mandible: MT1 Cases: ♂34.1 mm (±4.0) ♀34.2mm (±4.1) Controls: - Cranial base: NSe Cases: ♂40.8 mm (±3.1) ♀39.8mm (±2.9) Controls: - SBa Cases: ♂23.5 mm (±2.1) ♀23.2mm (±2.7) Controls: - NBa Cases:♂60.5 mm (±4.2) ♀60.1mm (±11.2) Controls: - NSBa Cases: ♂140.9º (±4.8) ♀139.6º (±4.6) Controls: -	Down Syndrome 4-6 months age group Maxilla: SNA Cases: ♂83.5º (±4.5) ♀83.6º (±3.8) Controls: - SpP Cases: ♂25,9 mm (±3.4) ♀25.4mm (±1.8) Controls: - Mandible: MT1 Cases: ♂38.3 mm (±4.2) ♀37.3mm (±3.1) Controls: - Cranial base: NSe Cases: ♂44.1 mm (±3.1) ♀43.2mm (±2.7) Controls: - SBa Cases: ♂26.1 mm (±3.0) ♀25.4mm (±2.6) Controls: - NBa Cases:♂65.4 mm (±4.6) ♀61.9mm (±3.9) Controls: - NSBa Cases: ♂137.8º (±5.4) ♀138.7º (±4.4) Controls: -	Down Syndrome7-12 months age group Maxilla: SNA Cases: ♂83.0º (±5.9) ♀83.4º (±3.5) Controls: - SpP Cases: ♂28.6 mm (±3.0) ♀27.6mm (±2.4) Controls: - Mandible: MT1 Cases: ♂40.3 mm (±3.1) ♀39.5mm (±3.2) Controls: - Cranial base: NSe Cases: ♂46.2 mm (±2.9) ♀44.6mm (±2.4) Controls: - SBa Cases: ♂27.7 mm (±2.2) ♀26.9mm (±2.2) Controls: - NBa Cases:♂68.6 mm (±3.8) ♀66.6mm (±3.5) Controls: - NSBa Cases: ♂136.3º (±8.1) ♀137.5º (±4.5) Controls: -		Down Syndrome 0-3 months age group Go Cases: ♂133.2º (±6.2) ♀134.1º (±6.1) MT2Cases: ♂19.7 mm (±2.6) ♀20.0mm (±3.2) Controls: - N-SpP Cases:♂20.1 mm (±2.0) ♀24.0mm (±3.1) Controls: - Down Syndrome 4-6 months age group Go Cases: ♂131.8º (±16.9) ♀132.8º (±5.0) Controls: - MT2 Cases: ♂23.7mm (±3.2) ♀23.0mm (±2.5) Controls: - N-SpP Cases: ♂22.9 mm (±3.0) ♀25.4mm (±1.0) Controls: - Down Syndrome 7-12 months age group Go Cases: ♂132.1º (±6.9) ♀130.1º (±5.8) Controls: - MT2 Cases: ♂25.5 mm (±2.5) ♀25.3mm (±2.9) Controls: - N-SpP Cases: ♂24.1 mm (±2.4) ♀27.6mm (±2.4) Controls: - Down Syndrome 13-18 months age group Go Cases: ♂130.5º (±5.5) ♀128.95º (±5.3) Controls: - MT2 Cases: ♂27.6 mm (±2.6) ♀27.2mm (±2.9) Controls: - N-SpP Cases: ♂26.6mm (±2.2) ♀30.0mm (±1.8) Controls: -		-	Class III with midface hypoplasia	OMIM #190685
									Down Syndrome 13-18 months age group Maxilla: SNA Cases: ♂82.9º (±4.5) ♀83.6º (±4.6) Controls: - SpP Cases: ♂30.8 mm (±2.4) ♀30.0mm (±1.8) Controls: - Mandible: MT1 Cases: ♂42.1 mm (±4.8) ♀43.8mm (±8.1) Controls: - Cranial base: NSe Cases: ♂48.6 mm (±2.8) ♀47.1mm (±2.8) Controls: - SBa Cases: ♂28.7 mm (±2.0) ♀28.1mm (±2.2) Controls: - NBa Cases:♂72.1 mm (±3.5) ♀69.9mm (±3.1) Controls: - NSBa Cases: ♂136.9º (±5.2) ♀136.5º (±4.8) Controls: -	Down Syndrome 19-24 months age group Maxilla: SNA Cases: ♂83.2º (±4.0) ♀84.2º (±3.9) Controls: - SpP Cases: ♂31.3 mm (±2.0) ♀30.8mm (±2.1) Controls: - Mandible: MT1 Cases: ♂43.8 mm (±3.4) ♀43.8mm (±5.3) Controls: - Cranial base: NSe Cases: ♂49.4 mm (±2.9) ♀48.4mm (±3.1) Controls: - SBa Cases: ♂29.5 mm (±2.6) ♀28.9mm (±2.3) Controls: - NBa Cases:♂73.2 mm (±4.6) ♀71.8mm (±3.8) Controls: - NSBa Cases: ♂135.7º (±4.8) ♀135.7º (±5.2) Controls: -	Down Syndrome 3 year old group Maxilla: SNA Cases: ♂82.8º (±3.7) ♀83.6º (±4.3) Controls: - SpP Cases: ♂31.8 mm (±1.9) ♀30.8mm (±2.1) Controls: - Mandible: MT1 Cases: ♂46.3 mm (±3.7) ♀45.7mm (±3.5) Controls: - Cranial base: NSe Cases: ♂50.5 mm (±3.1) ♀48.8mm (±3.3) Controls: - SBa Cases: ♂30.2 mm (±2.3) ♀30.4mm (±9.5) Controls: - NBa Cases:♂75.2 mm (±4.1) ♀72.8mm (±4.1) Controls: - NSBa Cases: ♂135.6º (±4.8) ♀136.0º (±5.3) Controls: -		Down Syndrome 19-24 months age group Go Cases: ♂132.3º (±9.2) ♀129.1º (±5.6) MT2 Cases: ♂29.6 mm (±3.6) ♀29.4mm (±2.8) Controls: - N-SpP Cases:♂27.6 mm (±2.8) ♀26.0mm (±4.1) Controls: - Down Syndrome 3 year old group Go Cases: ♂130,0º (±5,7) ♀127,7º (±16.1) Controls: - MT2 Cases: ♂30,5mm (±3,1) ♀30,4mm (±3,9) Controls: - N-SpP Cases: ♂29.4 mm (±3.1) ♀27.9mm (±3.0) Controls: - Down Syndrome 4 year old group Go Cases: ♂130,0º (±5,2) ♀127.7º (±6.0) Controls: - MT2 Cases: ♂33.1 mm (±3.6) ♀32.8mm (±3.6) Controls: - N-SpP Cases: ♂31.5 mm (±3.0) ♀30.6mm (±2.2) Controls: - Down Syndrome 5 year old group Go angle Cases: ♂129.3º (±5.4 ♀127.7º (±6.5) Controls: - MT2 Cases: ♂34.8 mm (±3.4) ♀35.4mm (±3.8) Controls: - N-SpP Cases: ♂32.1 mm (±2.4) ♀32.0mm (±2.6) Controls: -				
									Down Syndrome 4 year old group Maxilla: SNA Cases: ♂83.2º (±3.7) ♀82.9º (±4.8) Controls: - SpP Cases: ♂31.3 mm (±2.0) ♀30.8mm (±2.1) Controls: - Mandible: MT1 Cases: ♂49.7 mm (±3.8) ♀49.3mm (±3.4) Controls: - Cranial base: NSe Cases: ♂52.2 mm (±3.2) ♀50.9mm (±2.5) Controls: - SBa Cases: ♂31.0 mm (±2.3) ♀30.9mm (±2.4) Controls: - NBa Cases:♂77.3 mm (±4.3) ♀76.3mm (±3.8) Controls: - NSBa Cases: ♂135.4º (±5.0) ♀137.0º (±4.6) Controls: -	Down Syndrome 5 year old group Maxilla: SNA Cases: ♂83.0º (±3.6) ♀83.5º (±3.9) Controls: - SpP Cases: ♂33.5 mm (±3.6) ♀33.2mm (±2.5) Controls: - Mandible: MT1 Cases: ♂51.3 mm (±4.7) ♀51.3mm (±7.1) Controls: - Cranial base: NSe Cases: ♂52.8 mm (±2.9) ♀51.6mm (±3.1) Controls: - SBa Cases: ♂31.7 mm (±2.6) ♀31.3mm (±2.5) Controls: - NBa Cases:♂78.7 mm (±3.8) ♀77.4mm (±3.6) Controls: - NSBa Cases: ♂135.6º (±4.3) ♀136.1º (±5.5) Controls: -	Down Syndrome 6 year old group Maxilla: SNA Cases: ♂82.9º (±3.4) ♀82.6º (±3.3) Controls: - SpP Cases: ♂35.8 mm (±2.2) ♀34.4mm (±2.1) Controls: - Mandible: MT1 Cases: ♂54.3 mm (±8.4) ♀52.7mm (±3.4) Controls: - Cranial base: NSe Cases: ♂53.7 mm (±2.9) ♀51.8mm (±3.2) Controls: - SBa Cases: ♂32.9 mm (±2.5) ♀31.7mm (±2.4) Controls: - NBa Cases:♂80.8 mm (±4.3) ♀78.5mm (±3.5) Controls: - NSBa Cases: ♂136.0º (±4.9) ♀137.8º (±4.5) Controls: -		Down Syndrome 6 year old age group Go Cases: ♂127.7º (±5.3) ♀126.1º (±6.1) MT2 Cases: ♂36.4 mm (±3.1) ♀36.6mm (±4.1) Controls: - N-SpP Cases:♂33.8 mm (±2.6) ♀32.8mm (±2.1) Controls: - Down Syndrome 7-8 year old group Go Cases: ♂127.9º (±6.3) ♀126.0º (±6.0) Controls: - MT2 Cases: ♂38.6mm (±4.2) ♀38.1mm (±4.3) Controls: - N-SpP Cases: ♂36.4 mm (±3.4) ♀34.8mm (±3.2) Controls: - Down Syndrome 11-12 year old group GoCases: ♂123.0º (±6.1) ♀122.6º (±5.1) Controls: - MT2 Cases: ♂44.3 mm (±3.9) ♀46mm (±8.8) Controls: - N-SpP Cases: ♂40,0 mm (±3.5) ♀39.2mm (±3.1) Controls: - Down Syndrome 13-14 year old group Go Cases: ♂123.7º (±8.4) ♀121.5º (±3.7) Controls: - MT2 Cases: ♂47.2 mm (±5.3) ♀44.9mm (±5.4) Controls: - N-SpP Cases: ♂40,9 mm (±4.4) ♀39.2mm (±2.7) Controls: -				
									Down Syndrome 7–8 year old group Maxilla SNA Cases: ♂82.1º (±7.6) ♀83.1º (±4.1) Controls: - SpP Cases: ♂36.7 mm (±4.7) ♀35.1mm (±2.6) Controls: - Mandible: MT1 Cases: ♂55.8 mm (±5.1) ♀55.2mm (±3.9) Controls: - Cranial base: NSe Cases:♂54.8 mm (±3.4) ♀53.1mm (±3.2) Controls: - SBa Cases: ♂34.3 mm (±2.9) ♀32.9mm (±3.8) Controls: - NBa Cases: ♂83.3 mm (±5.0) ♀81.3mm (±5.7) Controls: - NSBa Cases: ♂136.8º (±5.4) ♀137.3º (±5.1) Controls: -	Down Syndrome 9-10 year old group Maxilla: SNA Cases: ♂81.0º (±8.5) ♀82.7º (±4.2) Controls: - SpP Cases: ♂36.3 mm (±2.0) ♀36.1mm (±3.0) Controls: - Mandible: MT1 Cases: ♂59.6 mm (±3.5) ♀60.6mm (±3.8) Controls: - Cranial base: NSe Cases: ♂55.5 mm (±3.1) ♀54.3mm (±3.1) Controls: - SBa Cases: ♂36.5 mm (±2.8) ♀35.4mm (±2.7) Controls: - NBa Cases:♂86.6 mm (±4.1) ♀84.8mm (±4.0) Controls: - NSBa Cases: ♂137.2º (±5.1) ♀137.7º (±4.4) Controls: -	Down Syndrome 11-12 year old group Maxilla: SNA Cases: ♂83.9º (±4.2) ♀83.6º (±3.1) Controls: - SpP Cases: ♂38.4 mm (±2.6) ♀37.3mm (±3.9) Controls: - Mandible: MT1 Cases: ♂63.6 mm (±3.0) ♀61.1mm (±4.2) Controls: - Cranial base: NSe Cases: ♂56.7 mm (±3.2) ♀54.9mm (±2.3) Controls: - SBa Cases: ♂37.0 mm (±3.2) ♀37.0mm (±2.2) Controls: - NBa Cases:♂88.0 mm (±4.4) ♀86.1mm (±4.2) Controls: - NSBa Cases: ♂135.5º (±6.8) ♀136.8º (±5.1) Controls: -		Down Syndrome13-14 year old group Maxilla SNA Cases: ♂82.8º (±4.4) ♀79.9º (±3.3) Controls: - SpP Cases:♂39.9 mm (±4.9) ♀37.3mm (±4.0) Controls: - Mandible: MT1 Cases: ♂66.0 mm (±6.3) ♀61.8mm (±1.5) Controls: - Cranial base: NSe Cases: ♂53.2 mm (±3.2) ♀53.2mm (±5.7) Controls: - SBa Cases:♂37.3 mm (±3.0) ♀36.2mm (±2.3) Controls: - NBa Cases: ♂89.3 mm (±5.1) ♀88.5mm (±5.2) Controls: - NSBa Cases: ♂136.9º (±5.9) ♀142.7º (±5.8) Controls: -				

♂ Male, ♀ Female, y years, m months, s syndrome.

Please see Supplementary Table 3 for the linear and angular measurements used on Table 1 and their definition.

**Table 2:** Genotyped Syndromes sharing the skeletal class III phenotype [Search strategy #Step1 and #Step2 results].

OMIM Syndrome	Gene	Loci	General phenotype	Craniofacial phenotype	References	Syndrome reference
Apert Syndrome	FGFR2	10q26.13	Apert syndrome is a congenital disorder characterized primarily by craniosynostosis, midface hypoplasia, and syndactyly of the hands and feet with a tendency to fusion of bony structures. Most cases are sporadic, but autosomal dominant inheritance has been reported	Head- Acrobrachycephaly - Turribrachycephaly - Large fontanel - Late-closing fontanel - Megalencephaly Face- High, broad forehead - Flat face - Midface hypoplasia - Mandibular prognathism Ears- Hearing loss - Chronic otitis media - Abnormal semicircular canals Eyes- Shallow orbits - Hypertelorism - Downslanting palpebral fissures - Proptosis Nose- Depressed nasal bridge - Choanal stenosis or atresia - Strabismus Mouth- Narrow palate - Cleft palate - Bifid uvula Teeth- Malocclusion - Delayed dental eruption	Mantilla-Capacho, J. M., Arnaud, L., Diaz-Rodriguez, M., Barros-Nunez, P. Apert syndrome with preaxial polydactyly showing the typical mutation Ser252Trp in the FGFR2 gene. Genet. Counsel. 16: 403-406, 2005.	OMIM #101200
Crouzon Syndrome	FGFR2	10q26.13	Crouzon syndrome is an autosomal dominant disorder characterized by craniosynostosis causing secondary alterations of the facial bones and facial structure. Common features include hypertelorism, exophthalmos and external strabismus, parrot-beaked nose, short upper lip, hypoplastic maxilla, and a relative mandibular prognathism	Head- Craniosynostosis - Brachycephaly Face- Frontal bossing - Maxillary hypoplasia - Mandibular prognathism Ears- Conductive hearing loss - Atretic external auditory canals Eyes- Optic atrophy - Shallow orbits - Proptosis - Hypertelorism - Strabismus - Exposure conjunctivitis/keratitis - Poor vision Nose- Parrot-like nose Mouth- Lateral palatal swellings Teeth- Dental crowding	Reardon, W., Winter, R. M., Rutland, P., Pulleyn, L. J., Jones, B. M., Malcolm, S. Mutations in the fibroblast growth factor receptor 2 gene cause Crouzon syndrome. Nature Genet. 8: 98-103, 1994. Glaser, R. L., Jiang, W., Boyadjiev, S. A., Tran, A. K., Zachary, A. A., Van Maldergem, L., Johnson, D., Walsh, S., Oldridge, M., Wall, S. A., Wilkie, A. O. M., Jabs, E. W. Paternal origin of FGFR2 mutations in sporadic cases of Crouzon syndrome and Pfeiffer syndrome. Am. J. Hum. Genet. 66: 768-777, 2000.	OMIM #123500
Achondroplasia	FGFR3	4p16.3	Achondroplasia is the most frequent form of short-limb dwarfism. Affected individuals exhibit short stature caused by rhizomelic shortening of the limbs, characteristic facies with frontal bossing and midface hypoplasia, exaggerated lumbar lordosis, limitation of elbow extension, genu varum, and trident hand	Skull- Jugular bulb dehiscence (in some patients) - Foramen magnum stenosis Head- Frontal bossing - Megalencephaly Face- Midface hypoplasia Ears- Recurrent otitis media in infancy and childhood - Conductive hearing loss Nose- Low nasal bridge	Bellus, G. A., Hefferon, T. W., Ortiz de Luna, R. I., Hecht, J. T., Horton, W. A., Machado, M., Kaitila, I., McIntosh, I., Francomano, C. A. Achondroplasia is defined by recurrent G380R mutations of FGFR3. Am. J. Hum. Genet. 56: 368-373, 1995.	OMIM #100800
Ectodermal Dysplasia 1, Hypohidrotic, X-Linked; Xhed	EDA	Xq13.1	Hypohidrotic, or anhidrotic, ectodermal dysplasia (HED/EDA) is characterized by a triad of signs comprising sparse hair (hypotrichosis), abnormal or missing teeth (anodontia or hypodontia), and inability to sweat (anhidrosis or hypohidrosis). Typical clinical manifestations also include dryness of the skin, eyes, airways, and mucous membranes presumably due to the defective development of several exocrine glands. Hypohidrotic ectodermal dysplasia can be associated with dysmorphic features (forehead bumps, rings under the eyes, everted nose, and prominent lips) and occasionally with absent nipples. Ectodermal dysplasia-1, due to mutation in the EDA gene, is the most frequent form of hypohidrotic ectodermal dysplasia	Head- Small cranial length Face - Frontal bossing - Hypoplastic maxilla - Small chin - Small facial height - Prominent supraorbital ridges Eyes- Periorbital wrinkles - Periorbital hyperpigmentation - Absent tears - Absent miebomian glands - Scant-absent eyebrows - Scant-absent eyelashes Nose - Small nose - Hypoplastic alae nasi - Nasal mucosa atrophy - Ozena - Depressed nasal root and bridge (‘saddle nose’) Mouth- Decreased palatal depth - Prominent lips Teeth- Hypodontia - Adontia - Microdontia - Conical teeth - Taurodontism	Cluzeau, C., Hadj-Rabia, S., Jambou, M., Mansour, S., Guigue, P., Masmoudi, S., Bal, E., Chassaing, N., Vincent, M.-C., Viot, G., Clauss, F., Maniere, M.-C., and 11 others. Only four genes (EDA1, EDAR, EDARADD, and WNT10A) account for 90% of hypohidrotic/anhidrotic ectodermal dysplasia cases. Hum. Mutat. 32: 70-77, 2011.	OMIM #305100
Trichodontoosseous syndrome	DLX3	17q21.33	Trichodentoosseous syndrome is an autosomal dominant disorder with complete penetrance characterized by abnormalities involving hair, teeth, and several bone structures.	Head- Dolichocephaly Face- Frontal bossing Teeth- Thin enamel - Small, widely spaced teeth - Teeth pits - Taurodontism - Periapical abscesses	Nguyen, T., Phillips, C., Frazier-Bower, S., Wright, T. Craniofacial variations in the tricho-dento-osseous syndrome. Clin. Genet. 83: 375-379, 2013.	OMIM #190320
Cleidocranial dysplasia, forme fruste, with brachydactyly; Cleidocranial dysplasia, forme fruste, dental anomalies only ; Cleidocranial dysplasia	RUNX2 RUNX2 RUNX2	6p21.1 6p21.1 6p21.1	The main clinical features of Cleidocranial dysplasia include persistently open skull sutures with bulging calvaria, hypoplasia or aplasia of the clavicles permitting abnormal facility in apposing the shoulders, wide pubic symphysis, short middle phalanx of the fifth fingers, dental anomalies, and often vertebral malformation.	Skull- Wormian bones - Bossing of frontal bone - Bossing of occipital bone - Bossing of parietal bone - Calvarial thickening - Absent frontal sinuses - Absent paranasal sinuses - Hypoplastic frontal sinuses - Hypoplastic paranasal sinuses - Large foramen magnum Head- Delayed fontanel closure - Parietal bossing - Anterior fontanel open in adults Face- Frontal bossing - Metopic groove - Midface hypoplasia - Micrognathia Ears- Deafness Eyes- Hypertelorism Nose- Low nasal bridge Mouth- Cleft palate - Narrow, high-arched palate Teeth- Delayed eruption of deciduous teeth - Delayed eruption of permanent teeth - Supernumerary teeth - Retention cysts - Enamel hypoplasia	Mundlos, S. Cleidocranial dysplasia: clinical and molecular genetics. J. Med. Genet. 36: 177-182, 1999.	OMIM #119600
Klinefelter syndrome	47 XXY	-	Klinefelter Syndrome (KS) is characterized by an extreme heterogeneity in its clinical and genetic presentation. Associations between clinical phenotype and genetic background are not yet completely understood; KS patients are traditionally as having tall stature, small testes, gynecomastia in late puberty, gynoid aspect of hips (broad hips), sparse body hair, signs of androgen deficiency and low serum testosterone coupled with elevated gonadotropins, and finally azoospermia, oligospermia with hyalinization and fibrosis of the seminiferous tubules	Face- mandibular prognathism, cleft lip, hemifacial microtia	M. Bonomi, V. Rochira, D. Pasquali, G. Balercia, E. A. Jannini, A. Ferlin & On behalf of the Klinefelter ItaliaN Group (KING), Klinefelter syndrome (KS): genetics, clinical phenotype and hypogonadism, Journal of Endocrinological Investigation volume 40, pages123–134(2017)	MCID:HYP 730
Down Syndrome	-	21q22.3	Down syndrome, the most frequent form of mental retardation caused by a chromosomal aberration, is characterized by well-defined and distinctive phenotypic features and natural history. The clinical presentation of DS is complex and variable. A few features occur to some degree in every individual with trisomy 21, including characteristic facial dysmorphology, a small and hypocellular brain, and the histopathology of Alzheimer disease, which is present by the fourth decade. Individuals with DS are invariably cognitively impaired, though the severity is highly variable. Hypotonia occurs frequently in newborns, and most have atypical dermatoglyphic features, though the specific subset of these is again individually variable.	Face- midface deficiency, mandibular prognathism, depressed nasal bridge, slanting eyes with epicanthic folds, ocular hypotelorism, and strabismus.	Epstein, C. J. Down syndrome, trisomy 21. In: Scriver, C. R.; Beaudet, A. L.; Sly, W. S.; Valle, D. (eds.): Metabolic Basis of Inherited Disease. New York: McGraw-Hill (pub.) 1989. Pp. 291-326. Roper, Randall J Reeves, Roger H, Understanding the basis for Down syndrome phenotypes, Journal of Developmental Biology, 2019, 7, 2.	OMIM #130650

### Quality evaluation and risk of bias in individual studies

Two investigators (M.C.F.T. and A.I.L) assessed the quality of the methodology of the selected studies. Disagreement was checked by an independent reviewer (A.V.C). The Newcastle-Ottawa scale (NOS) for case control studies was used to assess the quality of the selected studies. The NOS scale has 3 principal categories: initial assessment and selection of participants, comparability of groups, and assessment of the outcome of interest (Table 3).^45^ Studies with five or more points were considered high quality.^46^ A detailed explanation of the quality assessment of the included studies can be found in Table 3.

**Table 3:** Risk of bias assessment based on The Newcastle-Ottawa scale.

Reference	Selection				Comparability	Exposure			Total
	**1**	**2**	**3**	**4**	**5**	**6**	**7**	**8**	
Kreiborg S et al.^56^	A*	A*	B	A*	A**	A*	A*	A*	8
Lu X et al.^49^ (1)	A*	B	C	B	A**	A*	A*	A*	6
Lu X et al.^50^	A*	B	C	B	A**	A*	A*	A*	6
Reitsma HJ et al.^14^	A*	A*	A*	A*	A**	A*	A*	A*	9
Engel M et al. (2018)	A*	A*	C	A*	A**	A*	A*	A*	8
Lu X et al.^52^	A*	B	C	A*	A**	A*	A*	A*	7
Cardoso R et al. (2010)	A*	A*	C	C	A**	A*	A*	A*	7
Arslan S G et al. (2006)	A*	A*	B	A*	A**	A*	A*	A*	8
Sonnesen L et al.^54^	A*	A*	B	A*	A**	A*	A*	A*	8
Lexner MO et al. (2007)	A*	A*	C	C	A**	A*	A*	A*	7
Johnson EL et al.^48^	A*	A*	A*	A*	A**	A*	A*	A*	9
Nguyen T et al.^21^	A*	B	C	A*	A**	A*	A*	A*	7
Jensen BL et al.^22^	A*	A*	B	A*	A**	A*	A*	A*	8
Brkic H et al.^47^	A*	A*	B	A*	A*	A*	A*	A*	7
Ingerslev C H et al.^32^	A*	A*	B	A*	A*	A*	A*	A*	7
Brown T et al.^33^	A*	A*	B	A*	A*	A*	A*	A*	7
Babic M et al. (1992)	A*	A*	B	A*	A**	A*	A*	A*	8
Suri S et al.^27^	A*	A*	A*	A*	A**	A*	A*	A*	9
Korayem MA et al.^30^	A*	A*	B	A*	A**	A*	A*	A*	8
Silva Jesuino FA et al.^26^	A*	A*	B	A*	A**	A*	A*	A*	8
Clarkson C et al.^29^	A*	B	A*	A*	A*	A*	A*	A*	7
Fischer-Brandies H.^28^	A*	A*	C	A*	A**	A*	A*	A*	8

Total score calculated by the sum of the stars (*).

1. Is the case definition adequate?

(a) yes, with independent validation*.

(b) yes, e.g., record linkage or based on self-reports.

(c) no description.

2. Representativeness of the cases.

(a) consecutive or obviously representative series of cases.

(b) potential for selection biases or not stated.

3. Selection of controls.

(a) community controls*.

(b) hospital controls.

(c) no description.

4. Definition of controls.

(a) no history of disease (endpoint)*.

(b) no description of source.

Comparability.

5. Comparability of cases and controls on the basis of the design or analysis.

(a) study controls for personal factors*.

(b) study controls for any additional factor psychosocial factors*.

Exposure.

6. Ascertainment of exposure.

(a) secure record (e.g., surgical records)*.

(b) structured interview where blind to case/control status*.

(c) interview not blinded to case/control status.

(d) written self-report or medical record only.

(e) no description.

7. Same method of ascertainment for cases and controls.

(a) yes*.

(b) no.

8. Non-response rate.

(a) same rate for both groups.

(b) non respondents described.

(c) rate different and no designation.

Total score calculated by the sum of the stars (*).

### Meta-analysis of the data and summary measures

The meta-analysis of the data across the syndromes with underlying SCIII was performed with Epidat 3.1 (*Software for epidemiological analysis of tabulated data. Open and free version. Consellería de Sanidade Xunta de Galicia. WHO*). Four summary measures were assessed in the meta-analysis (i) The midface component was explored through two measurements: The angle formed by Sella-Nasion-point A (SNA); the linear measurement from the apex of the anterior nasal spine, or Spinal point, to the intersection between the nasal floor and the posterior contour of the maxilla, or Pterygomaxillary point (sp-pm); (ii) The lower face component was evaluated through two parameters: the angle formed by Sella-Nasion and point B (SNB); the angle formed by Sella-Nasion and Pogonion (SNPg). A random effect maximum likelihood meta-analysis was performed, forest plots were obtained, and heterogeneity was calculated using the Q statistic at a significance level of 10%.

## Results

**Fig. 1:**
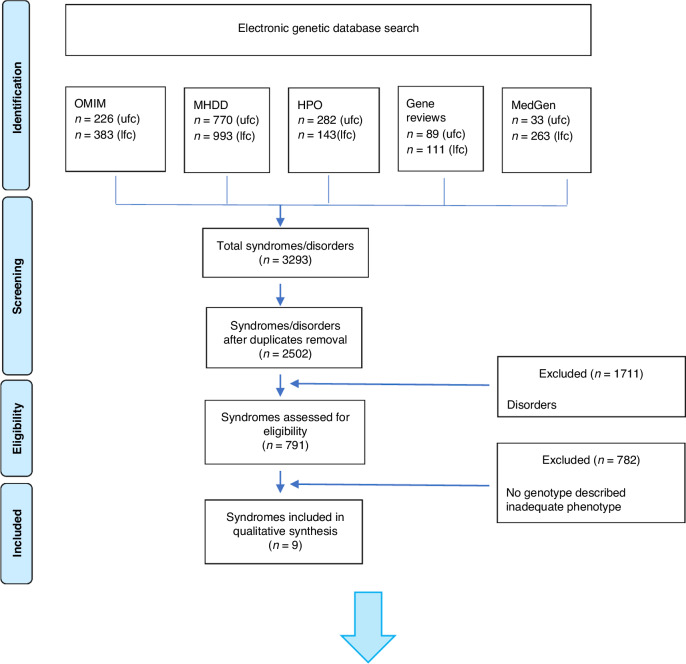
Flow chart diagram. The diagram illustrates the results from #Step 1 concerning the upper (ufc) and lower face component (lfc) and results from #Step 2 concerning the electronic database search.

The electronic search in *Online Mendelian Inheritance in Man* database (*OMIM), MalaCards Human Disease Database* (MHDD), *Human Phenotype Ontology* (HPO), GeneReviews and MedGen databases [Step 1#] (Fig. 1) identified 350 syndromes with a craniofacial phenotype compatible with skeletal class III related to prognathism, and 441 syndromes with a craniofacial phenotype compatible with SCIII related to maxillary hypoplasia, representing a total of 791 syndromes.

The electronic and manual searches defined in [Step 2#] identified 122 relevant studies. After full-text assessment for eligibility, 100 papers were rejected because they did not meet the criteria of this investigation. A total of 22 articles were selected and analyzed (Table 1).

The *Kappa index* score for inter-rater reliability for the electronic database search was 0.87, which is considered a strong level of agreement.

### Description of the individual characteristics of the studies

Overall results are summarized in Table 1. The articles were published between 1978 and 2020. Sample sizes ranged between 9 and 1896 patients. The selected samples included both male and female subjects in 12 of the 22 studies, exclusively male subjects in 4 studies^32,33,47,48^ and sex was not reported in 6 studies.^26,29,49–52^

Twenty-one of the included studies were published in English and one in Spanish,^29^ and represented data from different world regions, including North America,^22,27,48–50,52^ South America,^15,26,29,49,52^ Saudi Arabia,^30^ China,^49,52^ and several European countries.^20,22,28,32,33,47,51,53–56^

### Quality evaluation and risk of bias in individual studies

Our scoping review retrieved twenty-two articles. The Newcastle-Ottawa scale (NOS) for observational studies was used to assess the risk of bias in individual studies. A maximum score of eight points was awarded to studies that fulfilled 8/9 quality criteria. In our systematic search, all the selected studies scored 6 or more points (Table 3). Previous categorization studies considered studies with five points or more as high quality studies.^46,57,58^

### Identification of the particular syndromes specifically affected by the skeletal class III phenotype

**Fig. 2:**
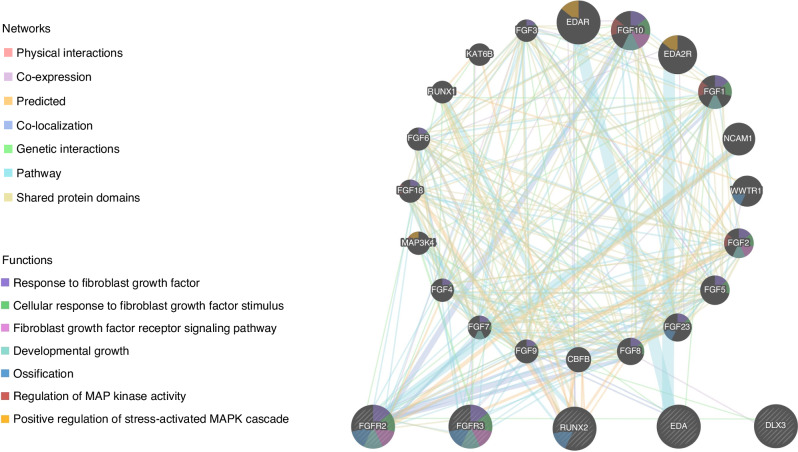
Genes related to syndromic skeletal Class III. See related info at genetic network analysis report. Network analysis report generated with Gene Mania software available at https://genemania.org.

Eight syndromes derived from [Step 1#] were finally selected for the present review. These were Apert syndrome (AS) (OMIM#101200); Crouzon syndrome (CS) (OMIM#123500); achondroplasia (ACH (OMIM#100800); X-linked hypohidrotic ectodermal dysplasia (XLHED) (OMIM#305100); tricho-dento-osseous syndrome (TDO) (OMIM#190320); cleidocranial dysplasia (CCD) (OMIM#119600); Klinefelter syndrome (KS) (MCID: HYP730) and Down syndrome (DS) (OMIM#190685). These syndromes represent congenital conditions with a well-characterized genetic basis (Fig. 2). The available phenotypic and genotypic information is provided in Table 2.

#### Characterization of skeletal class III phenotype components of the selected syndromes: midface and lower face. (Supplementary Files 5, 6)

**Fig. 3:**
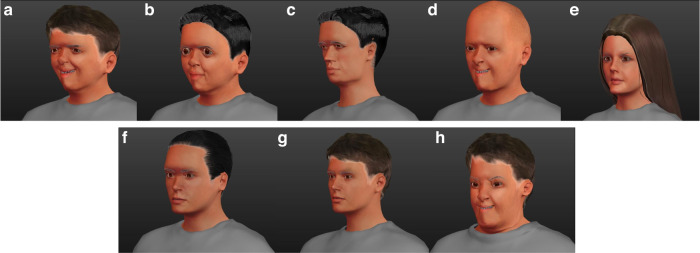
Craniofacial phenotype of genotypes syndromes exhibiting the skeletal class III phenotype.^2^ **a** Apert Syndrome; **b** Crouzon Syndrome; **c** achondroplasia; **d** X Linked Hypohidrotic ectodermal dysplasia; **e** trichodentoosseous syndrome; **f** cleidocranial dysplasia; **g** Klinefelter Syndrome; **h** Down Syndrome. Image obtained using Make Human software available at http://www.makehumancommunity.org.

The results derived from [Step 2#] enabled us to draw the following conclusions about the midface components of the selected syndromes (Fig. 3a–h):

##### Midface component

A. Two *craniosynostosis*-related syndromes [Apert syndrome (AS) and Crouzon syndrome (CS)] have a common clinical feature derived from upper maxillary hypoplasia. Cephalometric descriptions of AS patients included a retruded position of the maxilla and class III malocclusion of remarkable severity with very low SNA angle values, ranging between 64.7°–77.9°^51,55^ (Table 1). Similarly, CS patients exhibited midface retrusion with varying severity in both Caucasian [SNA = 75.65°]^52^ and Asian patients [SNA = 70.56]^52^ (Table 1). The midface retrusion observed in CS patients was similar in both ethnicities and resulted from the combined effect of hypoplastic size and backward displacement.^52^
  While comparing AS and CS patients a more severe SCIII phenotype was observed in AS patients, linked to reduced SNA and ANB angles.^51^
  Patients with CS,^51^ on the other hand, had larger SN/PP angles [SN/PP = −0.15°] than those with AS [SN/PP = −11.39°], suggesting an anterior rotation of the maxilla in relation to the cranial base (Table 1; Fig. 3a, b).
B. *Patients affected with achondroplasia (ACH)* simultaneously presented an adequately positioned maxilla in relation to the cranial base [SNA = 82.31°] and forward inclination of the maxilla.^15^ However, considering that the cranial base was reduced in both the posterior and anterior portions, we can infer that the N point was retropositioned and that the maxilla consequently assumed a more retracted position.^15^ In addition, the maxilla was smaller in the anteroposterior and transverse directions^15^ (Table 1; Fig. 3c).
C. *Patients with X-linked hypohidrotic ectodermal dysplasia (XLHED)* showed a shorter and more retrognathic maxilla^20,48,54^ in relation to the anterior cranial base [SNA = 76.4°]^20^ suggesting a SCIII pattern [ANB = −0.5°]^20^ related to midface hypoplasia (Table 1; Fig. 3d).
D. *Patients exhibiting tricho-dento-osseous syndrome (TDO)* were described as having a retrognathic maxilla in relation to the anterior cranial base [SNA = 78.84°],^21^ although the midface length [Co-A = 91.47 mm] was similar between subjects and controls.^21^ The decreased ANB angle further suggested a SCIII phenotype [ANB = 1.27°] (Table 1; Fig. 3e).^21^
E. Patients with *cleidocranial dysplasia* (CCD), presented a prognathic maxilla in relation to the cranial base [s-n-sp = 96.3°].^21^ This apparent protrusion may be related to the significantly reduced dimensions overall observed in the cranial base [s-n = 70.38 mm; s-ba = 44.1 mm],^21^ and the increased upward curvature of the clivus, conditioning the maxilla to assume a more anterior position (Table 1; Fig. 3f).
F. Patients with *syndromes related to chromosome aneuploidy*, particularly *Klinefelter syndrome (KS)* showed a reduced maxillary length [ANS-PNS = 55.0 mm]^21^ when compared to controls. Nevertheless, the maxilla assumed a prognathic position in the sagittal plane [SNA = 85.2°]^21^ and was almost parallel to the cranial base in syndromic patients [NS/SpP = 80.0°].^21^ (Table 1; Fig. 3g)

Likewise*, Down syndrome (DS) patients* showed a reduced maxillary length and midface hypoplasia, in five different studies.^26–30^ Nevertheless, the maxillary position relative to the cranial base was typically within the normal range^26–30^ [SNA ranging between 79.9°–82.47°^27^].

Essentially, patients with DS differed from the norm in terms of reduced maxillary length and cranial base length (Table 1; Fig. 3h). Furthermore, the significant overall reduction in craniofacial dimensions in DS patients that may explain less remarkable differences between syndromic and control groups.^26^

##### Lower face component

Results derived from the included studies about the lower face components enable us to draw the following conclusions about the characteristics of the mandible (Fig. 3a–i):

A. With respect to the *two craniosynostosis-related syndromes* (AS and CS), in AS patients, the sagittal analysis of the mandible revealed SNB angles ranging between 72.98°–78.35°, suggesting a relatively small mandible. Posterior facial height was also decreased [S-Go = 46.37 mm],^50^ inducing a backward rotation of the mandible in relation to the anterior cranial base and contributing to a brachycephalic phenotype (Table 1).^50^ The analysis of CS subjects showed that SNB angles were within the norm [SNB = 80.28°]^51^ and that effective mandibular length and mandibular body length were 8–10% shorter in Asian and Caucasian patients when compared to matched controls.^56^ There was 19% less mandibular volume in Asian CS patients (*p* = 0.102) and 15% less in Caucasian CS patients (*p* = 0.142).^52^ Prognathism in CS patients may, therefore, be related to displacement rather than to elongation of the mandible (Table 1; Fig. 3b). Interestingly, the Wits measurement showed a reduction of 10.17 mm in CS Asian patients and of 5.52 mm in Caucasian patients when compared to matched controls.^52^ The more severe mandibular protrusion of Asian versus Caucasian CS patients is probably related to the inherent morphological predisposition of Asians to prognathism.^52^
B. *ACH*-affected patients showed a protruding mandible in relation both to the cranial base and the maxilla [SNB = 83.22°; SND = 80.1°].^15^ The mandible presented a normal-sized ramus and reduced body length [,^15^ with a tendency towards a dolichofacial pattern (Table 1; Fig. 3c).^15^
C. *XLHED patients* exhibited an anteriorly inclined mandible in relation to the cranial base,^59^ and a prognathic mandible when compared to matched,^59^ which contributed greatly to the SCIII tendency [ANB = −0.5°].^20^ (Table 1; Fig. 3d).^48^
D. *TDO* patients were described as having a normally positioned mandible [SNB = 77.58°].^21^ Interestingly, mandibular body length was significantly longer in TDO subjects when compared to matched controls, with a mean difference of 3.6 mm. Ramus height [Ar-Go] was decreased and the gonial angle in the two groups was comparable. The relative mandibular prognathism (Fig. 3e) was reflected in the dentoalveolar structures by a smaller ANB angle (Table 1).^21^
E. *CCD patients* showed a markedly prognathic and anteriorly inclined mandible [s-n-pg = 90.93°].^22^ The sagittal jaw relationship indicated a SCIII relationship in both male and female CCD groups, being negative in the male group [ss-n-pg = −2.3°].^22^ In the vertical plane, there was a significant reduction of the jaw relationship [NL/ML = 11.4°] and the mean anterior facial height was reduced [n-gn = 113.1 mm].^22^ A reduced length of the mandible [pgn-cd] was observed in both male and female syndromic groups (Table 1; Fig. 3f).
F. *Syndromes related to chromosome aneuploidy* showed important differences concerning the lower face components. KS patients were described [50, 34] as having a prognathic [SNPg = 85.34°]^47^ and downward inclined mandibles [ML/MR = 127.70°].[34]. In addition, the mandibular ramus was notably shorter [(Ar-Tgo = 51.06 mm] when compared to matched controls (Table 1; Fig. 3g).^33^

With respect to **DS** patients, two studies^26,30^ described a decreased mandibular length associated with a smaller mandibular body and ramus.(32) Interestingly, the SNB angle was larger in syndromic subjects than in unaffected controls, and ranged between 78.4°–82.41°.^27^ Consistent with this, the relatively prognathic mandible^26,27^ and small retruded maxilla resulted in a smaller ANB and a decreased Wits appraisal value.^29^ DS patients also showed an anterior crossbite, a negative overjet, a reduced overbite and a tendency towards an anterior open bite (Table 1; Fig. 3h).^27^ (Supplementary Files 7, 8)

#### Chromosomal and genetic characterization of the particular syndromes that exhibit the skeletal class III phenotype

- Specific variations in the FGFR *genes*, particularly mutations on chromosome 10 [*FGFR2; 10q26.1]* have been linked to two craniosynostosis syndromes: AS and CS. FGFR-related craniosynostosis syndromes are autosomal dominant conditions and exhibit complete penetrance with variable expression.^60^ The same FRFR mutation can give rise to more than one syndromic phenotype and different mutations can produce the same syndromic phenotype.^60^ Consequently, AS and CS can be described as having a polygenic or multifactorial nature, probably with environmental influences.^60^
  FGFR mutations could influence maxillo-mandibular development through different FGF/FGFR signaling mechanisms:^61,62^ (i) in CS, the vast majority of mutations map to C342R and C278F, two residues that are critical for intramolecular disulfide bridge formation in the receptor subunit (Ig3), while the crosslinking of unpaired cysteines (C332Y, Y340H, C342Y, C342R, C342S, S35C, W290G, T341P, C278F) leads to covalent dimerization of receptor subunits in FGFR2 [OMIM*176943], resulting in their constitutive activation; (62),^63^ (ii) in AS, specific amino acid substitutions (S252W and P253R) in FGFR2 cause new or enhanced FGF binding affinities.(62),^63^
- Alterations on chromosome 4, particularly mutations in FGFR3;*4p16.3* are related to ACH.^64^ ACH, the most common type of short-limbed dwarfism, is an autosomal dominant disorder whose underlying mechanism is a defect in the maturation of the cartilage growth plate of long bones resulting from a gain-of-function mutation in FGFR3 [OMIM*134934].^65^ Unregulated signal transduction through FGFR3 leads to modifications of the bone phenotype, such as a distinctive facial appearance with midfacial hypoplasia and a more protrusive chin and mandible (Fig. 3c).^15^ More than 97% of ACH cases are caused by one of two mutations (G1138A and G1138C) in the FGFR3 gene that result in a specific amino acid substitution: G380R.^66^
- X chromosome variations, i.e., EDA mutations (Xq12-q13) have been related to the vast majority of hypohidrotic ectodermal dysplasia (HED) cases.^67,68^ Males are hemizygous and present the classical HED phenotype, while in the heterozygous female, the expression of symptoms varies considerably due to different levels of X-chromosome inactivation.^67–70^
  Despite the genotypic heterogeneity of XLHED, to date, the most prevalent mutations are located between exons 3 and 9 of EDA (94.4%).^71^ The classic phenotype of XLHED, with dental abnormalities, hypohidrosis, and craniofacial dysmorphologies (Fig. 3d), has been linked to mutated exons 5 and 6.^16^
- Variations in chromosome 17, particularly mutations at 17q21.33, are related to one particular syndrome exhibiting the SCIII phenotype: TDO. This locus is particularly associated with the distal-less homeobox protein, DLX3 [OMIM*600525], a member of the Distal-less (DLX) family.^72^ Mutations in the DLX3 gene are responsible for TDO in humans.^72^ The DLX3 gene plays an essential role in epidermal stratification and the development of ectodermal structures, such as hair, teeth and skeletal structures.^72^ Six DLX3 gene mutations have so far been identified in restricted family groups.^73–75^ The most common of these DLX3 mutations is a frameshift mutation (c.571_574delGGGG; G191RfsX66).^73^ DLX3 haploinsufficiency leads to disruption of essential regulatory mechanisms related with osteogenic signaling. (Fig. 3e).^76^
- Chromosome 4 also hosts core binding factor A1 [(CBFA1/RUNX2);*6p21.1]*. Mutations on this gene are related to CCD and cause open fontanels, hypoplasia of the clavicles, tooth abnormalities and the SCIII phenotype.^22^
  A total of 62 types of CBFA1/RUNX2 mutation have been identified to date in CCD patients, including deletions, insertions, nonsense and missense mutations, and changes in splicing sites.^77–80^ These mutations induce unregulated RUNX2 [OMIM*60211] activation, which might explain the severity of craniofacial phenotypes found in CCD patients, including mandibular prognathism (Fig. 3f).^22^
- Particular chromosome aneuploidies have been related to the SCIII phenotype, specifically syndromes exhibiting supernumerary X chromosomes,^81^ such as KS. Sex chromosomes, in addition to their primary role in gonadal differentiation, also play a key role in craniofacial development, morphology and size.^47^ The variability of the KS phenotype could be related to the extra X chromosome, influencing genome-wide gene expression^34^ and also epigenetic modifications such as DNA hypermethylation.^82^ The severity of mandibular prognathism in KS (Fig. 3g) may be correlated with the number of supernumerary X chromosomes.^81^

DS has the highest birth rate of all chromosomal abnormalities.^26–30^ Nevertheless, its gene expression mechanism is most likely complex and suggests a polygenic mechanism in which many genes interact to cause various disruptions in development in different parts of the craniofacial complex.^83^ The known surplus of genetic material in chromosomal pair 21 disrupts the normal development and results in generalized and localized growth disturbances, namely midface hypoplasia. (Fig. 3h; Table 2).

## Discussion

To the best of our knowledge, this is the first review to identify specific syndromes exhibiting a shared skeletal class III phenotype and simultaneously to gather valuable scientific information about the genetics of craniofacial development and malformation. This review further provides an update on the craniofacial characteristics, genetic etiology, molecular pathways (Fig. 2) and possible genotype-phenotype interactions on syndromic SCIII patients.

Using the evidence of a scoping review methodology,^41^ we designed an a priori protocol to define our research topic, objectives and methods. Nevertheless, scoping reviews do not assess the rigor or quality of studies as soundly performed as a systematic review approach does.^36,37^ Therefore, concluding results should be interpreted with caution in terms of their direct implications in the clinical field. Notwithstanding, in order to make a tentative systematic analysis, results from the selected studies were analyzed from a qualitative perspective, scoring 6 or more points in all the included studies. Therefore, the included studies might be considered as high-quality studies.^46,57,58^

It has been consistently documented that specific dentofacial dysmorphologies and syndromes are highly influenced by genetic factors and associated with moderate to high heritability.^84^ Conditions involving atypical craniofacial development may give a clearer picture of the genetic etiology of craniofacial development since several genes/gene regions involved in atypical (syndromic) patterns of craniofacial development may also be involved in normal-range craniofacial variation.^85^

To understand the pathophysiology of SCIII malocclusion, it is important to bear in mind that the craniofacial development takes place as a result of a “molecular dialog” between the cranial neural crest stem cells CNCSC and the epithelium in which several important signaling pathways and four main families of growth factors are involved.^13^ The specific pathways involved are the FGFR, HH, and WNT signaling pathways and the TGF-β signaling pathway, which includes BMPs and activins.^9–13^

The first major pathway involved in craniofacial development is the FGF signaling pathway.^8^

Of particular relevance are the mutations located on chromosome 10 at 10q26.13, more specifically, FGFR2 mutations related to AS and CS. These syndromes share various phenotypic features including the premature fusion of one or more cranial sutures, midfacial hypoplasia and/or relative mandibular prognathism,^8,86^ and are often associated with other skeletal and soft tissue abnormalities. Midfacial hypoplasia can be severe, but is variable within and across FGFR2-related craniosynostosis syndromes.^87^

According to our research, the most striking aspect of SCIII in FGFR2-related craniosynostosis syndromes is midface retrusion.^50–52^

Current literature has agreed on the underlying assumption that the abnormal craniofacial morphology is significantly more severe in AS than in CS.^12,56,88^

Various theories have been put forward to explain midface retrusion in craniosynostosis disorders, although these theories do not completely explain the observed phenotypes.^56^ It has been proposed that midfacial growth is primarily related to the cartilaginous growth centers and synchondrosis of the midline cranial base,^89^ and secondarily to orbital growth, the maxillary sinuses and alveolar process development.^89^ Different studies hypothesized^89,90^ that reduced development of the anterior and posterior cranial base could be related to synostosis of the coronal and spheno-frontal sutures, which would explain the absence of maxillary advancement. The spheno-occipital region would, on the other hand, tend to cause a shortening of the clivus. A shorter cranial base would move the mandibular condyle forward and a SCIII malocclusion would occur.^91^ The mandible, therefore, is not immune to growth changes in other areas. Consistent with this, the cephalometric evaluation of the mandible is heterogenous and ranges from “prognathic” to “normal”, and even includes some reports of “retrognathia”.^56,92,93^ Most descriptions are the result of qualitative clinical observations and few reports are based on quantitative measurements.^94^

The FGF signaling pathway may also be disrupted by alterations in chromosome 4, particularly FGFR3 mutations. These mutations are associated with ACH, the most prevalent dwarfism syndrome in humans.^15,95–98^ Unregulated signal transduction through FGFR3 results in inappropriate differentiation of the growth plate cartilage and abnormal long bone development^65^ The literature has highlighted relative mandibular prognathism, related to maxillary retropositioning,^15^ as a distinctive feature of ACH.^15,95–98^ Our results agree with those of previous studies indicating that mandibular prognathism is related to a smaller cranial base angle and to maxillary retropositioning.^15^ In addition, the mandible, is located in an anterior position with respect to the cranial base, contributing to the observed SCIII phenotype.

The second major signaling pathway, the TGFβ signaling pathway, may be substantially affected during development. Given the important role of TGFβ and BMPs during embryogenesis, mutations in genes involved in this signaling pathway can cause a wide range of skeletal and craniofacial dysmorphologies of varying degrees of severity.^99–101^ BMPs are strongly expressed during early craniofacial development.^102^ Functional interactions between BMP-MSX, WNT and ectodysplasin (EDA) have been described in NCCs, underlining the role of the EDA signaling pathway in craniofacial patterning and growth.^103^ Mutations in the EDA gene have been related to the most common form of ectodermal dysplasia, XLHED.^16^ Craniofacial dysmorphologies including mandibular prognathism and maxillary hypoplasia, have been reported in subjects with XLHED, although most studies are limited to case reports and a few cephalometric studies.^19,20,53,104^ According to our research, subjects with XLHED exhibit maxillary hypoplasia and mandibular prognathism during growth as shown by increased age dependent differences in the maxillo-mandibular dimensions.^48^ The angular measurements reflecting the sagittal position of the maxilla relative to the cranial base confirm this morphological tendency.^20,48,54^ While mandible body length decreased, it assumed a relatively prognathic position,^48^ as confirmed by the negative ANB angle.^20^ Overall, the cephalometric analysis of XLHED indicated a SCIII tendency.^20,48,54^

The patterning of embryonic ectoderm also depends on transcription factors such as Distal-less 3 (DLX3), which have different responses to BMP signaling in the ectoderm.^105^ DLX3 is essential for skeletal morphogenesis and acts as a scaffold for nucleic acids and regulatory factors involved in skeletal gene expression.^106^ In humans, mutations in DLX3 cause TDO. Although patients with TDO have been described as having protruding mandibles with obtuse gonial angles^107^ the selected comparison methods remain unclear. According to our results,^21^ TDO subjects exhibited a SCIII pattern characterized by reduced ANB and SNA angles.^21^ A retrusive maxilla aligned with a normal growing mandible gave rise to the observed SCIII phenotype.^21^ Our observations coincide with previous studies,^72^ in which 84% of TDO subjects and 80% of unaffected relatives had a smaller maxilla when compared to the Bolton standards. The literature however suggests,^21,72^ that the facial dysmorphologies observed in this syndrome could be familial traits independent of TDO. It is unclear whether the SCIII pattern is related to an inherited familial trait or to a mutation in the DLX3 gene.^21,72^

The WNT, BMP, FGF, SHH signaling pathways converge on regulatory factors such as RUNX2 that regulate epithelial‐mesenchymal interactions.^108^ These pathways come together to control maxillary and mandibular growth patterning components during development.^12,108^

Specific RUNX2 mutations are related to CCD. Unregulated RUNX2 activation may explain the severity of craniofacial phenotypes found in CCD patients,^25,109,110^ including the markedly prognathic mandible associated with a SCIII relationship and reduced anterior facial height.^22^

The third major molecular pathway concerning the regulation of craniofacial development is the WNT signaling pathway.^111^ WNT inactivation has been related to reduced growth of the facial processes and to maxillary hypoplasia.13,^112^ Furthermore, dysregulated WNT signaling has also been related to KS.^34^ KS is caused by an extra copy of the X chromosome, typically producing a 47 XXY karyotype.^34^ The hypothesis that an extra X chromosome causes mandibular prognathism and that the lack of an X-Chromosome causes mandibular retrognathism has been supported by several studies.^32,33,113,114^

Considering the results of cephalometric analyses of the Turner and Klinefelter syndromes as a whole,^31,33^ there is no doubt that the loss or the addition of an X chromosome affects craniofacial morphology. The consequence of the particular aneuploidy is obvious in the lateral facial profile, with increasing prognathism from 45X through 46 XX, and 46XY to 47XXY.^31,33^ According to our results, mandibular prognathism is observed both in relation to the cranial base and to the maxilla.^31–33,47^ In general, and compared to normal males, the additional X chromosome in those affected by KS appears to have a slightly depressant effect on the linear growth of the face, except for certain depth measurements such as mandibular corpus length, which is enhanced.^31^ Nonetheless, the specific way in which the XXY chromosome complex affects the craniofacial growth, including the possible role of hormones and the time of onset and duration of the disturbances, remains a matter of conjecture.^28^

The fourth key regulatory signaling pathway is mediated by the HH family of genes. In this context, craniofacial developmental studies have shown that the Sonic Hedgehog (SHH) gene is expressed in the ectoderm of the frontonasal and maxillary processes during early development.^12^ Abnormal SHH activation has been related to DS.^35,115^

The characteristic craniofacial phenotype of DS has been linked to reduced responsiveness of CNC cells to SHH signaling, resulting in newborn syndromic infants exhibiting disproportionately small skulls.^116^

Our results show that the relation of the sagittal position of the maxilla to the cranial base (SNA) did not change significantly during growth and did not differ from matched controls.^26,28^ Furthermore, our observations are in accordance with previous craniofacial growth studies^28,117^ where the effective maxillary length, the palatal plane length and the vertical dimension of the nasomaxillary complex were diminished. Despite the maxillary hypoplasia, the evolution over time of the effective maxillary length and the palatal plane length was similar in DS patients and matched controls.^127^ This means that although patients with DS start from an initial situation of deficiency, the maxillomandibular discrepancy remains fairly stable over time. Thus, DS patients during the prepubescent, pubescent and postpubescent periods seem to grow at a similar rate to the general population.^127^

However, our research found controversial results concerning the mandible of DS patients. One study^30^ found no significant differences in mandibular measurements between DS subjects and matched controls, whereas two others^26,27^ found that the mandible was smaller in DS subjects. Despite these observations, the included studies were in agreement with the assumption that a Class III malocclusion often develops in DS children^26,27,29,30^ and remains into adulthood.^29^ The small retruded maxilla and relatively prognathic mandible led to a smaller ANB^26,27,29,30^ and a decreased Wits appraisal.^29,30^

It is unlikely that the overall craniofacial differences between DS patients and controls are due to environmental effects, where a wider distribution of measurement scores would otherwise be expected. Hence, the expression of the genetic mechanism is most likely complex and suggests a polygenic mechanism in which many genes interact to cause various disturbances in different parts of the craniofacial complex. The known surplus of genetic material in the chromosomal pair 21, disturbs the normal polygenic model reflecting generalized and localized growth disturbances.^83^

### Clinical Relevance and final remarks

#### Scientific rationale for the study

Studies on craniofacial morphology have identified specific signaling molecules and transcription factors that bind to regulatory regions of the genome and regulate cell behavior, and consequently influence the process of craniofacial development.^12^ Craniofacial disorders and syndromes occur when mutations in the sequence of either a gene or a group of these genes alter the expression or function of the encoded proteins.^118^ Craniofacial dysmorphologies seem to be related more to subtle modifications in the highly coordinated program of cell division in the cranial mesenchyme than to basic patterning defects.^62^ Nevertheless, certain important aspects of craniofacial development are not yet completely understood. Defects in specific genes encoding growth factors or their receptors have been shown to be related to syndromic^62^ and non-syndromic craniofacial abnormalities.^8^

#### Main findings

This study provides cutting edge evidence on the cephalometric characteristics of genetically confirmed syndromes exhibiting SCIII malocclusion. Our results show that eight different syndromes exhibit the SCIII phenotype and that four major pathways regulating craniofacial development are affected in these syndromes, implying that they have a critical effect on this skeletal imbalance.

Although a simple correspondence in syndromic and non-syndromic patients is not anticipated, we hypothesize that more of the genes listed in our research could be involved in human craniofacial development, especially those concerning maxillary or mandibular development. Our results underline the need for further research on common genetic and molecular pathways in SCIII syndromes.

### Supplementary information

Supplementary File 1

Supplementary File 2

Supplementary File 3

Supplementary File 4

Supplementary File 5

Supplementary File 6

Supplementary File 7

Supplementary File 8

Supplementary File 9

## Data Availability

All supplementary data is available upon reasonable request to the corresponding author.
