# Supplementary material for: Craniofacial syndromes and class III phenotype: common genotype fingerprints? A scoping review and meta-analysis
Source: Pediatr Res. 2024 Feb 12;95(6):1455–75. doi: 10.1038/s41390-023-02907-5 (PMC11126392; doi:10.1038/s41390-023-02907-5)

-- Appert Syndrome: UPPER MAXILLA (SNA)--

Confidence level: 95,0%
Number of studies: 3
Sort by: Year
Sorting orientation: Ascending

HETEROGENEITY

Dersimonian and Laird's heterogeneity test

Q statistic (Chi-square)	df	p-value
492,0966	2	0,0000

Heterogeneity statistics	Estimator
Variance between studies	393,7930
Variance within studies	1,4620
Coefficient RI	0,9963
variance due to the variance between studies)	
Variation coeff. between studies	0,9497

(Proportion of total variance due to the variance between studies)

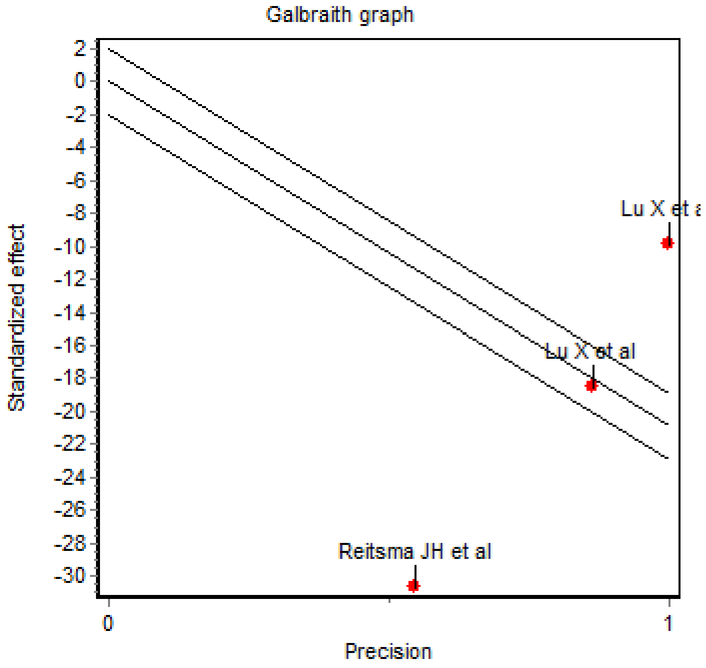

INDIVIDUAL AND COMBINED RESULTS

Study	Weights(%) Fixed eff.	Weights(%) Random eff.	Year	n	d	CI(95,0%)

Reitsma JH et al	2012	493	-56,1229	-59,7046	-52,5413	14,5931	33,2114
Lu X et al	2018	179	-21,4650	-23,7271	-19,2030	36,5859	33,3802
Lu X et al	2019	54	-9,9400	-11,8982	-7,9818	48,8210	33,4084
Fixed effects		726	-20,8961	-22,2643	-19,5278		
Random effects		726	-29,1251	-51,6343	-6,6159		

FOREST PLOT CUMULATIVE META-ANALYSIS(Random effects)

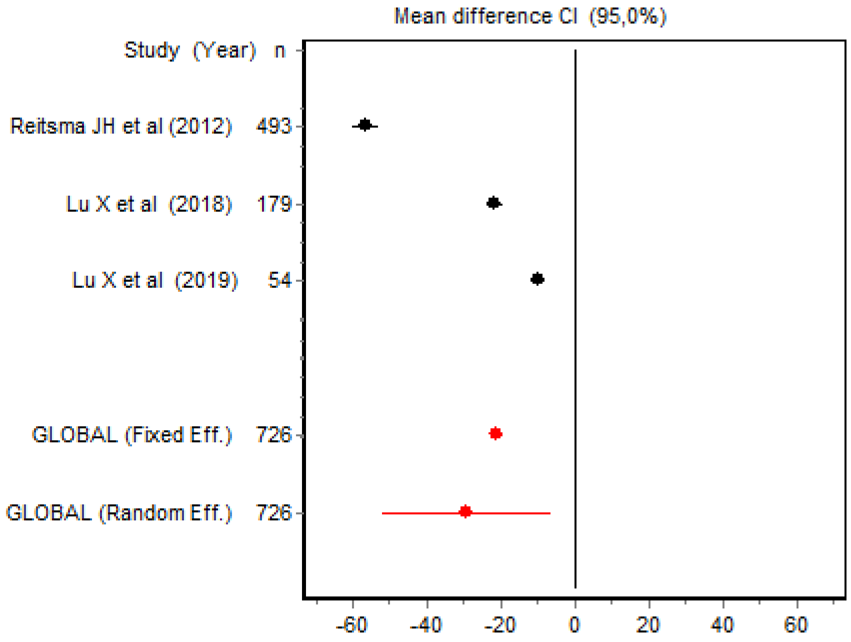

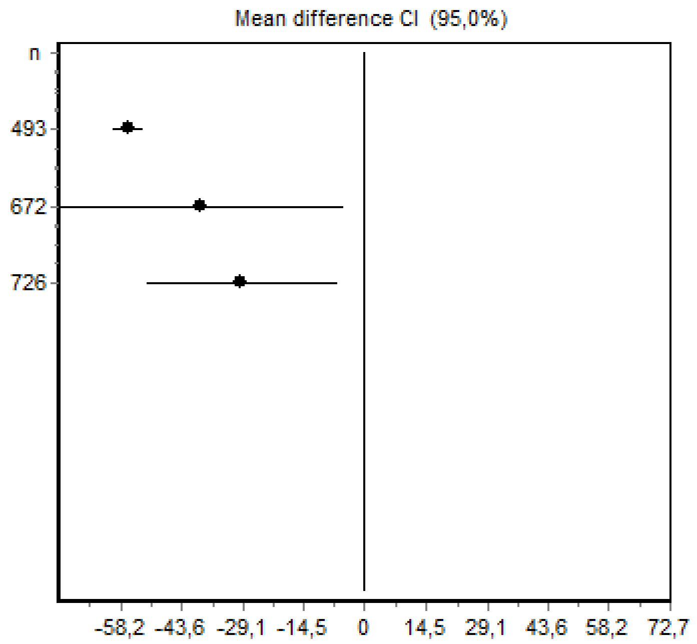


```
PUBLICATION BIAS

Begg test
Z statistic p-value
----------- -------
     1,0445  0,2963

Egger test
t statistic     df      p-value
----------- ------      -------
   -11,1351      1       0,0570
```

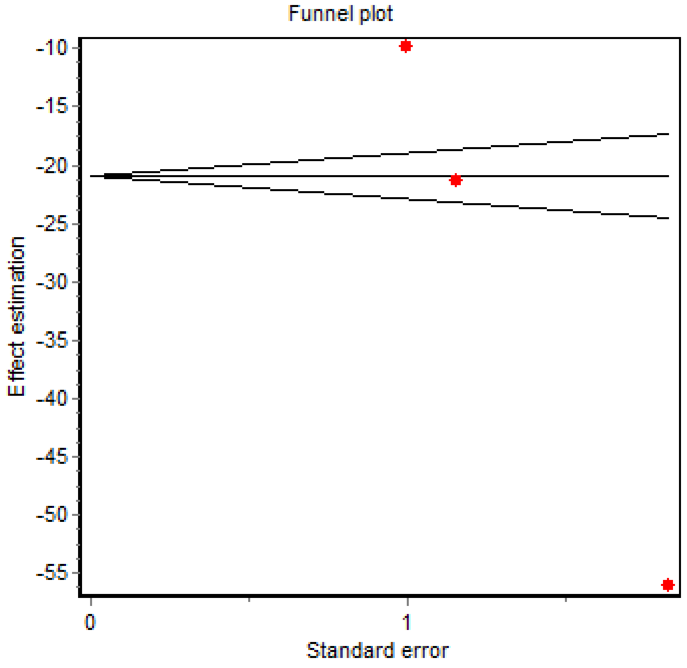

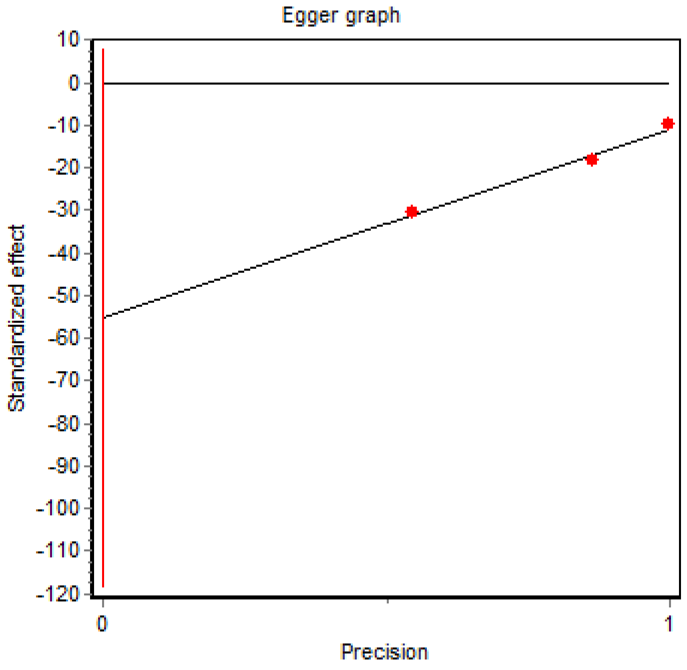

SENSITIVITY ANALYSIS

RANDOM EFFECTS MODEL

CI(95,0%)
Omitted study Year n d Lower limit Upper limit Relative change (%)

Reitsma JH et al	2012	233	-15,6881	-26,9823	-4,3938	-46,14
Lu X et al	2018	547	-33,0061	-78,2645	12,2522	13,33
Lu X et al	2019	672	-38,7650	-72,7291	-4,8010	33,10
GLOBAL		726	-29,1251	-51,6343	-6,6159	

Influence graph

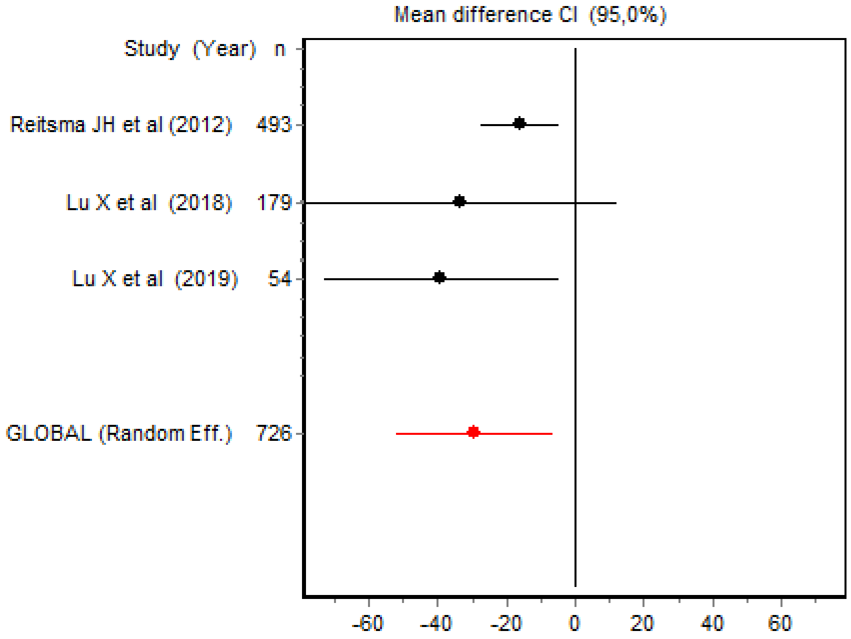

-- Down Syndrome: UPPER MAXILLA (SNA)--

```
  Confidence level:  95,0%
 Number of studies: 2
          Sort by: Year
Sorting orientation: Ascending
```

HETEROGENEITY

Dersimonian and Laird's heterogeneity test

Q statistic (Chi-square)	df	p-value
163,7720	1	0,0000

Heterogeneity statistics	Estimator
Variance between studies	17,8860
Variance within studies	0,1096
Coefficient RI	0,9939 (Proportion of total variance due to the variance between studies)
Variation coeff. between studies	3,2841

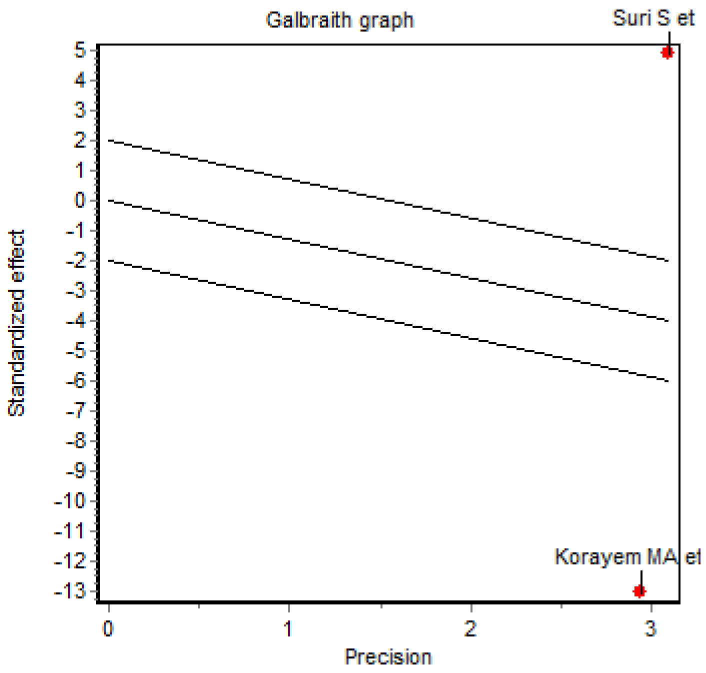

INDIVIDUAL AND COMBINED RESULTS

Study	Weights(%) Fixed eff.	Weights(%) Random eff.	Year	n	d	CI(95,0%)

```
      -------     -------
Suri S et al            2010      50      1,5629      0,9295      2,1962
      52,4838     50,0152
Korayem MA et al        2014     120     -4,4365     -5,1021     -3,7708
      47,5162     49,9848
---------------------- ----  ------      ------      ------      ------
      -------     -------
Fixed effects                    170     -1,2878     -1,7466     -0,8289
Random effects                   170     -1,4359     -7,3151     4,4433
```

FOREST PLOT CUMULATIVE META-ANALYSIS(Random effects)

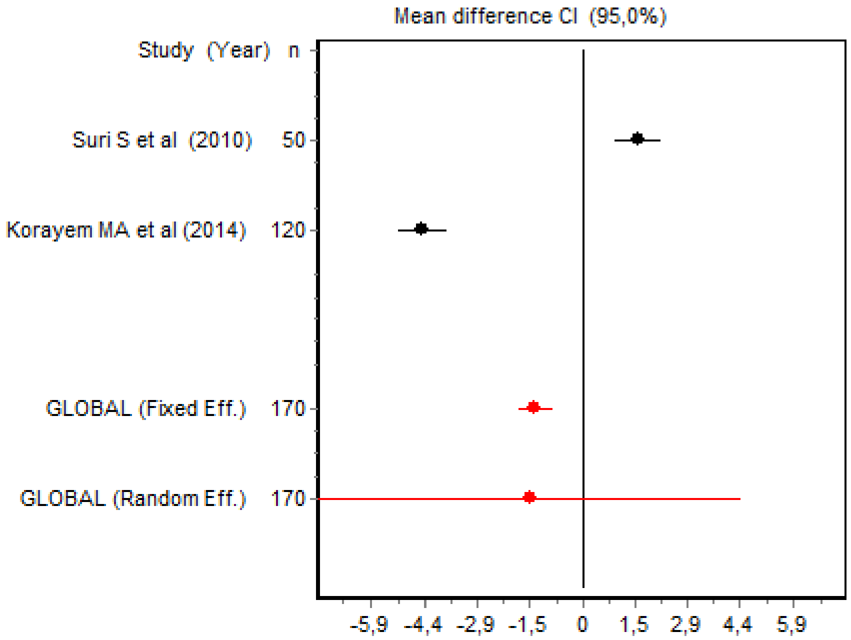

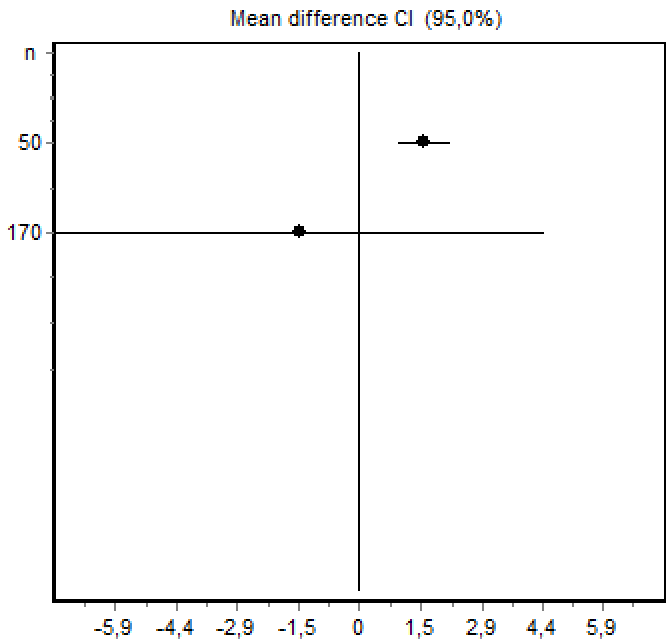

PUBLICATION BIAS

Begg test

Z statistic	p-value
0,0000	1,0000

-- Klinefelter Syndrome: UPPER MAXILLA (sp-pm)--

```
  Confidence level:  95,0%
 Number of studies: 3
          Sort by: Year
Sorting orientation: Ascending
```

HETEROGENEITY

Dersimonian and Laird's heterogeneity test

Q statistic (Chi-square)	df	p-value
176,1076	2	0,0000

Heterogeneity statistics	Estimator
Variance between studies	6,6535
Variance within studies	0,0691
Coefficient RI	0,9897

(Proportion of total variance due to the variance between studies)

Variation coeff. between studies	3,0319

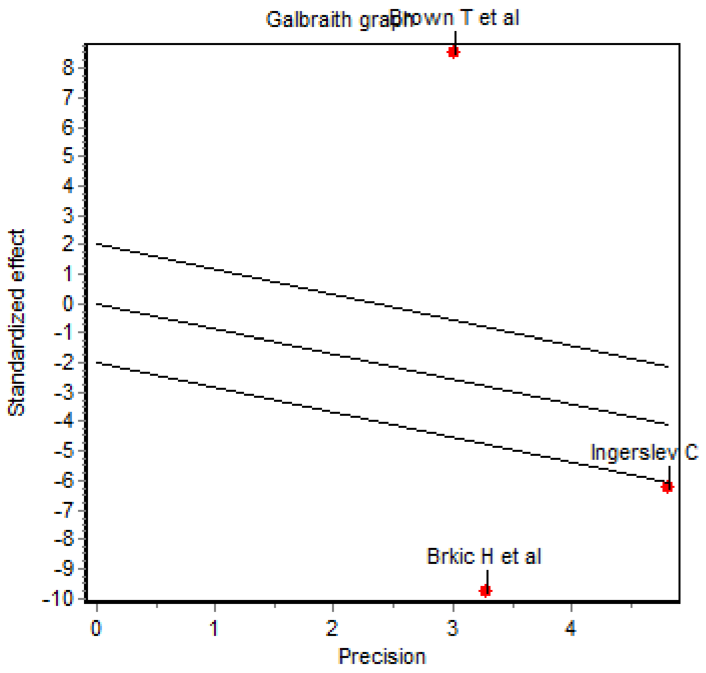

INDIVIDUAL AND COMBINED RESULTS

```
         Weights(%)
Study                  Year       n           d          CI(95,0%)
      Fixed eff.  Random eff.
```

Ingerslev C et al	1978	139	-1,2942	-1,7000	-0,8885	53,7828	33,5240
Brown T et al	1993	73	2,8037	2,1562	3,4512	21,1185	33,1954
Brkic H et al	1994	95	-2,9755	-3,5694	-2,3815	25,0986	33,2806
Fixed effects		307	-0,8508	-1,1483	-0,5532		
Random effects		307	-0,4934	-3,4300	2,4432		

FOREST PLOT CUMULATIVE META-ANALYSIS(Random effects)

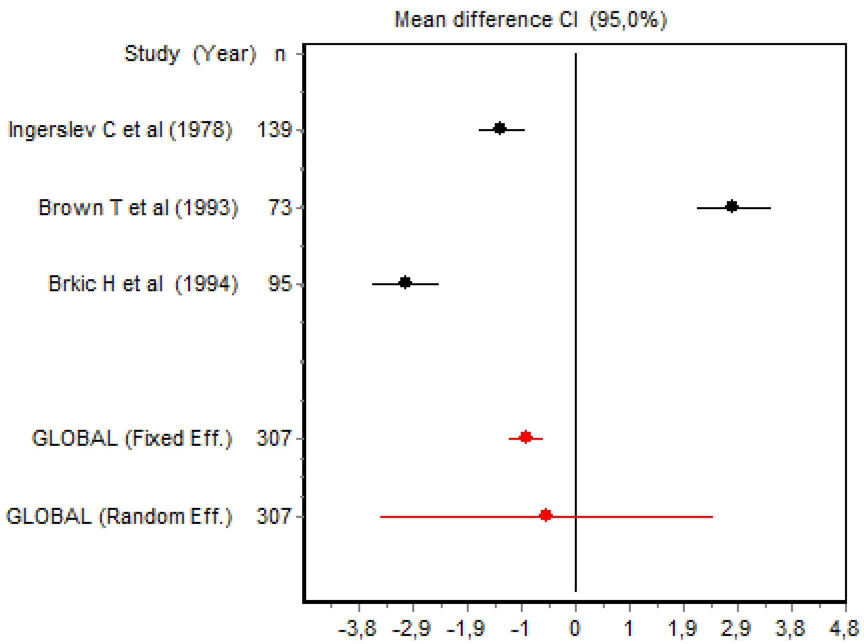

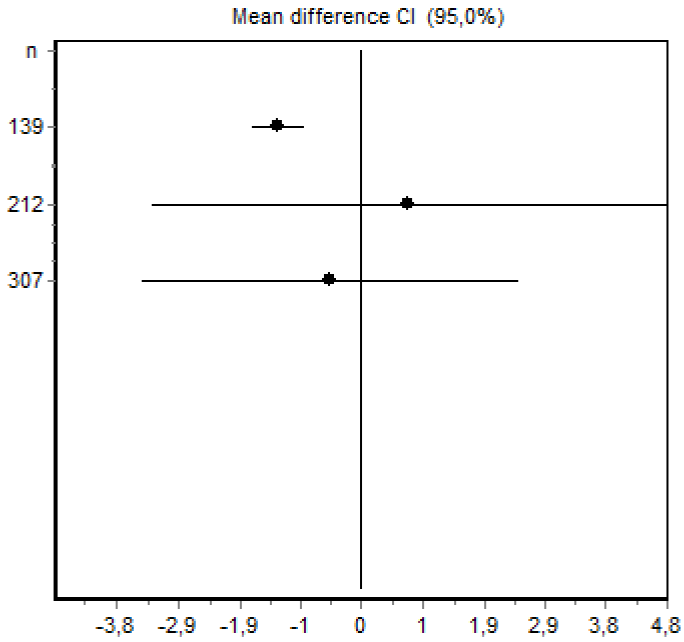

PUBLICATION BIAS

Begg test

Z statistic	p-value
0,0000	1,0000

Egger test

t statistic	df	p-value
0,4336	1	0,7395

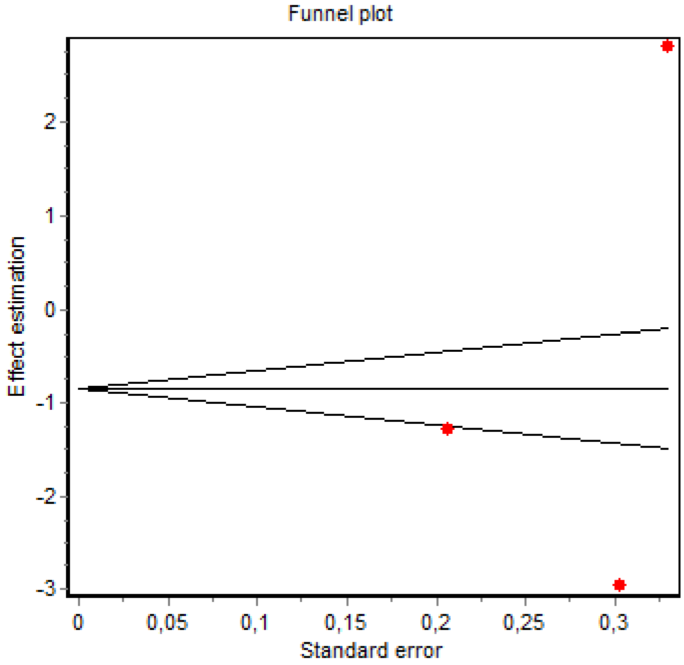

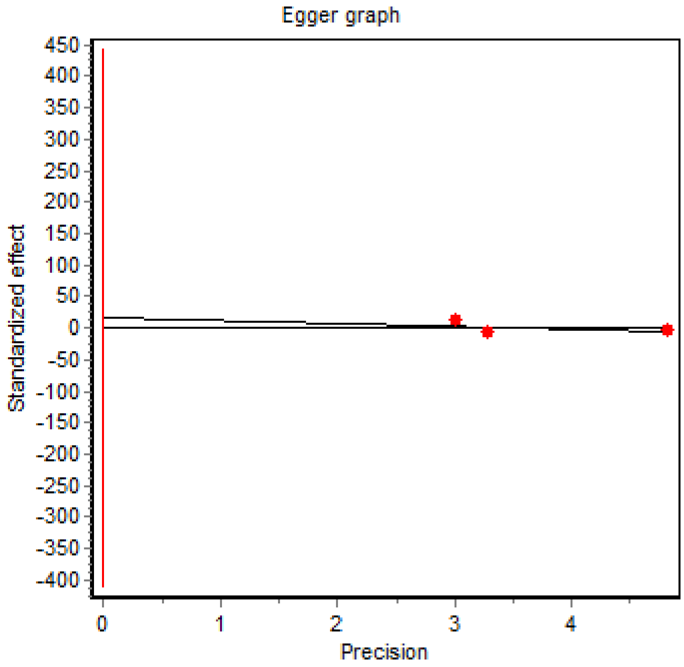

SENSITIVITY ANALYSIS

RANDOM EFFECTS MODEL

				CI(95,0%)		
Omitted study	Year	n	d	Lower limit	Upper limit	Relative change (%)

Ingerslev C et al	1978	168	-0,0874	-5,7508	5,5760	-82,29
Brown T et al	1993	234	-2,1203	-3,7676	-0,4729	329,70
Brkic H et al	1994	212	0,7466	-3,2692	4,7625	-251,31
GLOBAL		307	-0,4934	-3,4300	2,4432	

Influence graph

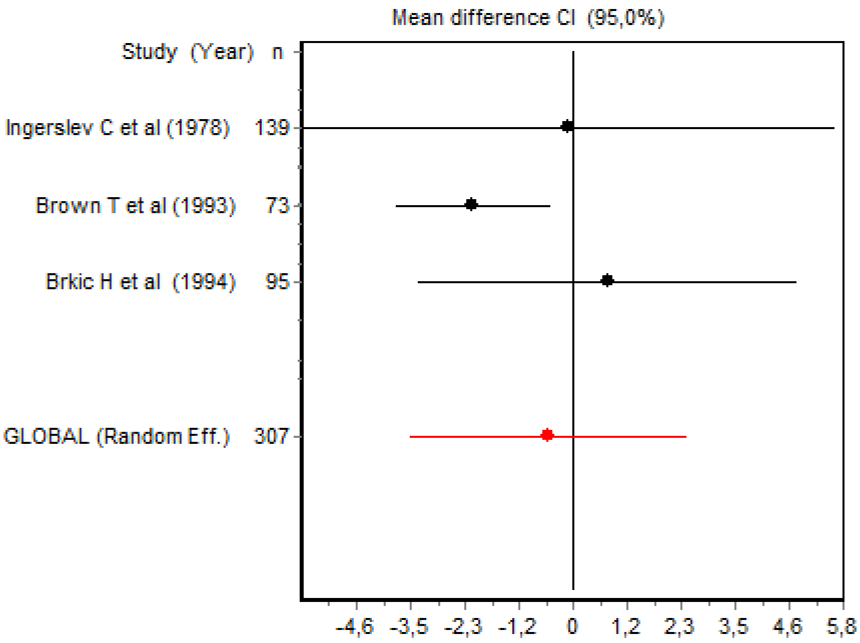

-- X Linked Hypohidrotic Ectoderrmal dysplasia: UPPER MAXILLA (SNA)--

Confidence level: 95,0%
Number of studies: 3
Sort by: Year
Sorting orientation: Ascending

HETEROGENEITY

Dersimonian and Laird's heterogeneity test

Q statistic (Chi-square)	df	p-value
289,8579	2	0,0000

Heterogeneity statistics	Estimator
Variance between studies	42,6642
Variance within studies	0,2787
Coefficient RI (Proportion of total variance due to the variance between studies)	0,9935
Variation coeff. between studies	0,7076

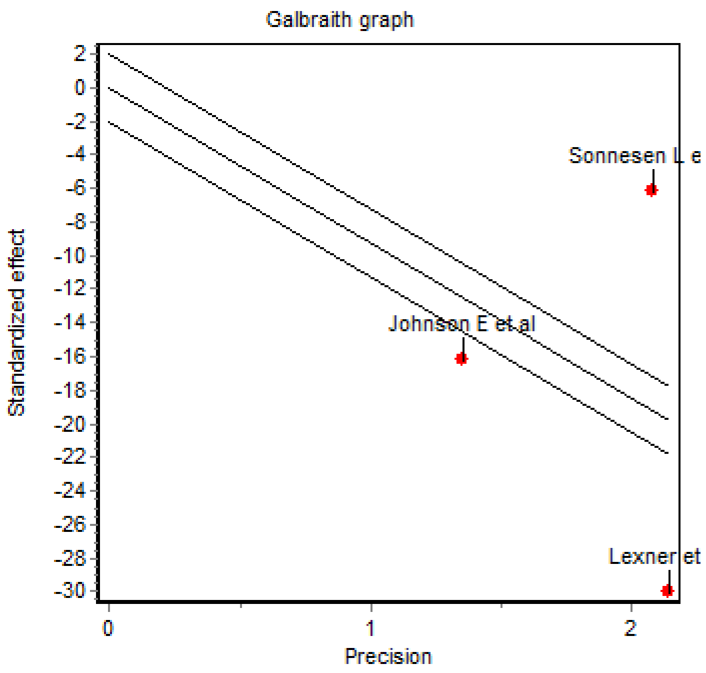

INDIVIDUAL AND COMBINED RESULTS

Weights(%)
Study Year n d CI(95,0%)
Fixed eff. Random eff.

Johnson E et al	2002	148	-11,9972	-13,4429	-10,5515	17,0722	33,1684
Lexner et al	2007	512	-14,0268	-14,9409	-13,1127	42,7000	33,4210
Sonnesen L et al	2017	37	-2,9647	-3,9065	-2,0229	40,2278	33,4106
Fixed effects		697	-9,2303	-9,8276	-8,6329		
Random effects		697	-9,6577	-17,0775	-2,2379		

FOREST PLOT CUMULATIVE META-ANALYSIS(Random effects)

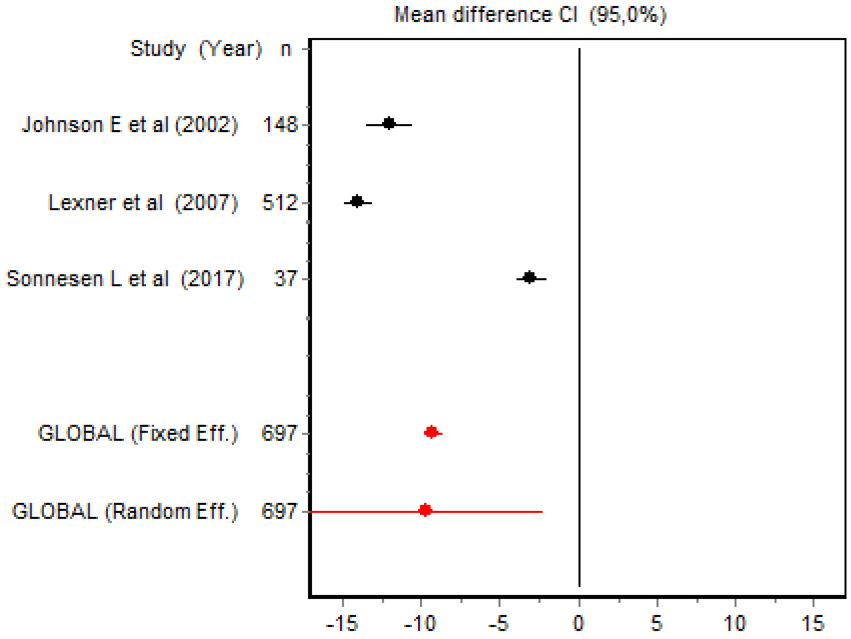

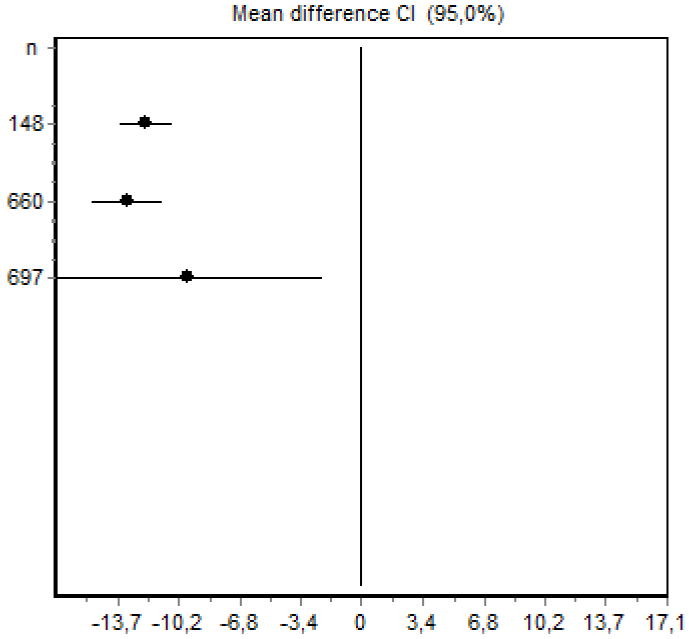

PUBLICATION BIAS

Begg test

Z statistic	p-value
0,0000	1,0000

Egger test

t statistic	df	p-value
-0,1818	1	0,8855

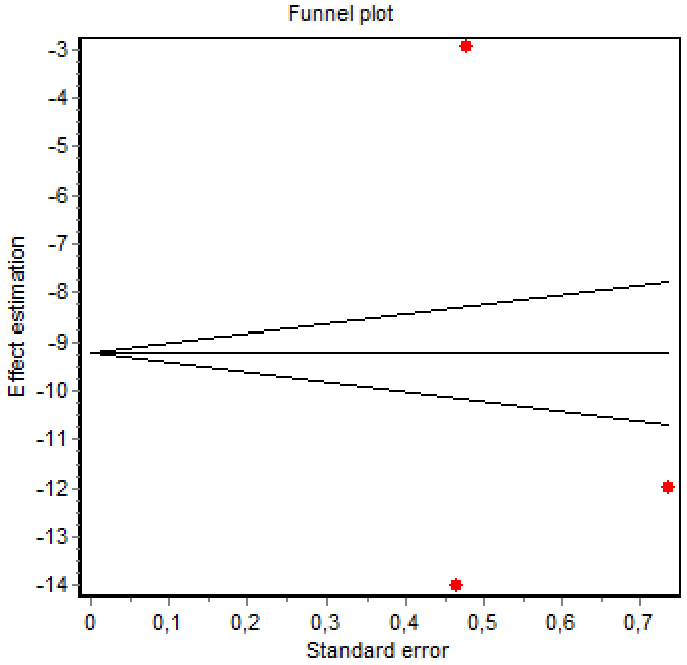

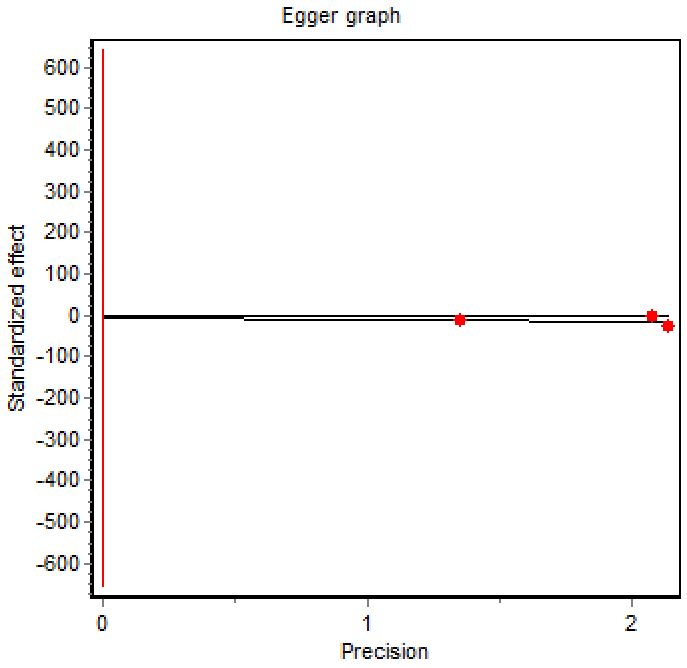

SENSITIVITY ANALYSIS

RANDOM EFFECTS MODEL

CI(95,0%)
Omitted study Year n d Lower limit Upper limit Relative change (%)

Johnson E et al	2002	549	-8,4964	-19,3370	2,3443	-12,03
Lexner et al	2007	185	-7,4636	-16,3152	1,3880	-22,72
Sonnesen L et al	2017	660	-13,0924	-15,0752	-11,1097	35,56
GLOBAL		697	-9,6577	-17,0775	-2,2379	

Influence graph

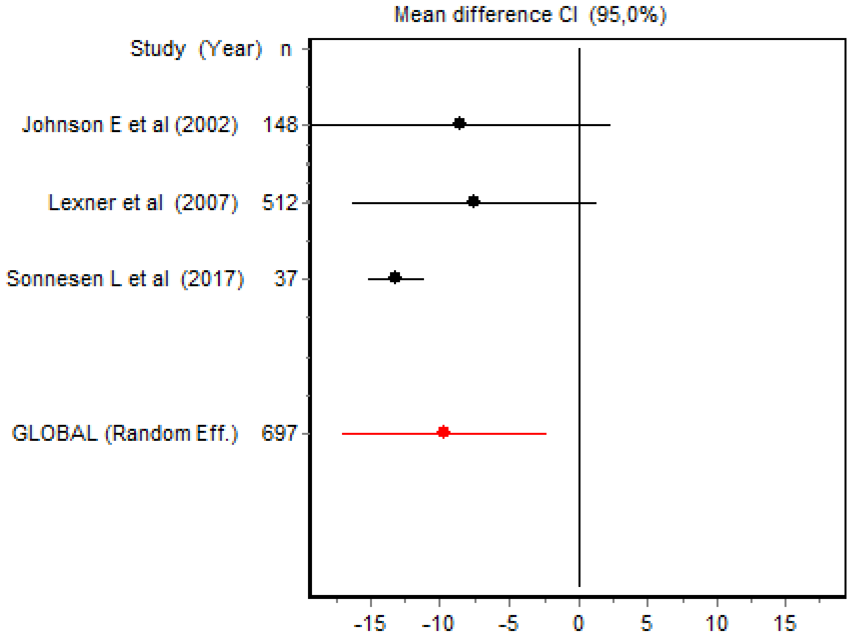

Supplement: Supplementary file 7 [file 41390_2023_2907_MOESM7_ESM.pdf]
