# Supplementary material for: Craniofacial syndromes and class III phenotype: common genotype fingerprints? A scoping review and meta-analysis
Source: Pediatr Res. 2024 Feb 12;95(6):1455–75. doi: 10.1038/s41390-023-02907-5 (PMC11126392; doi:10.1038/s41390-023-02907-5)

## -- Appert Syndrome: LOWER MAXILLA (SNB)--

Confidence level: 95,0%
Number of studies: 3
Sort by: Year
Sorting orientation: Ascending

HETEROGENEITY

Dersimonian and Laird's heterogeneity test

| Q statistic (Chi-square) | df | p-value |
|---|---|---|
| 411,9636 | 2 | 0,0000 |

| Heterogeneity statistics | Estimator |
|---|---|
| Variance between studies | 48,3395 |
| Variance within studies | 0,2249 |
| Coefficient RI | 0,9954 (Proportion of total variance due to the variance between studies) |
| Variation coeff. between studies | 3,5292 |

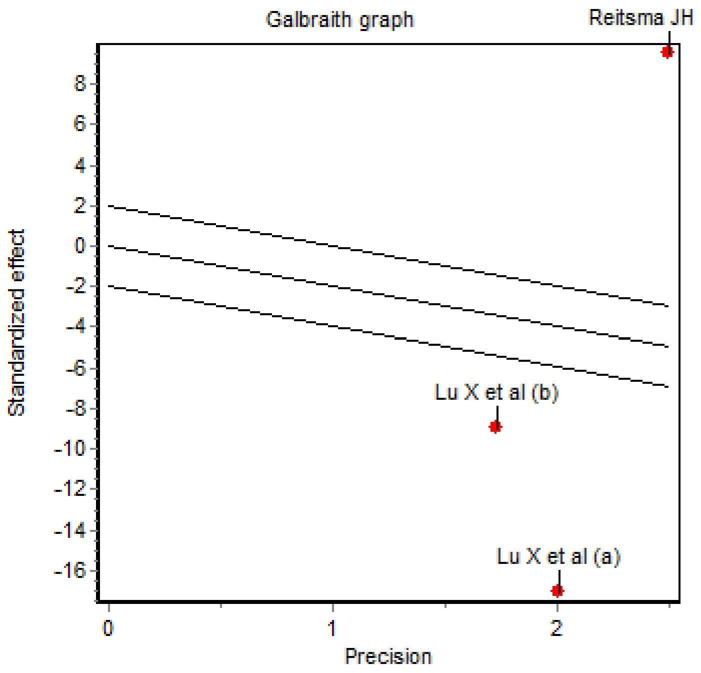


INDIVIDUAL AND COMBINED RESULTS

| Study | Year | n | d | CI(95,0%) | Weights(%) Fixed eff. | Weights(%) Random eff. |
|---|---|---|---|---|---|---|

| | | | | | | | |
|---|---|---|---|---|---|---|---|
| --------------------- | ---- | ------ | ------ | ------ | ------ | ------- | ------- |
| Reitsma JH et al | 2012 | 493 | 3,7926 | 3,0098 | 4,5754 | 46,9938 | 33,3926 |
| Lu X et al (b) | 2019 | 54 | -5,1717 | -6,2993 | -4,0441 | 22,6463 | 33,2749 |
| Lu X et al (a) | 2019 | 179 | -8,5017 | -9,4756 | -7,5278 | 30,3598 | 33,3325 |
| --------------------- | ---- | ------ | ------ | ------ | ------ | ------- | ------- |
| Fixed effects | | 726 | -1,9700 | -2,5066 | -1,4334 | | |
| Random effects | | 726 | -3,2883 | -11,1758 | 4,5992 | | |

FOREST PLOT CUMULATIVE META-ANALYSIS(Random effects)

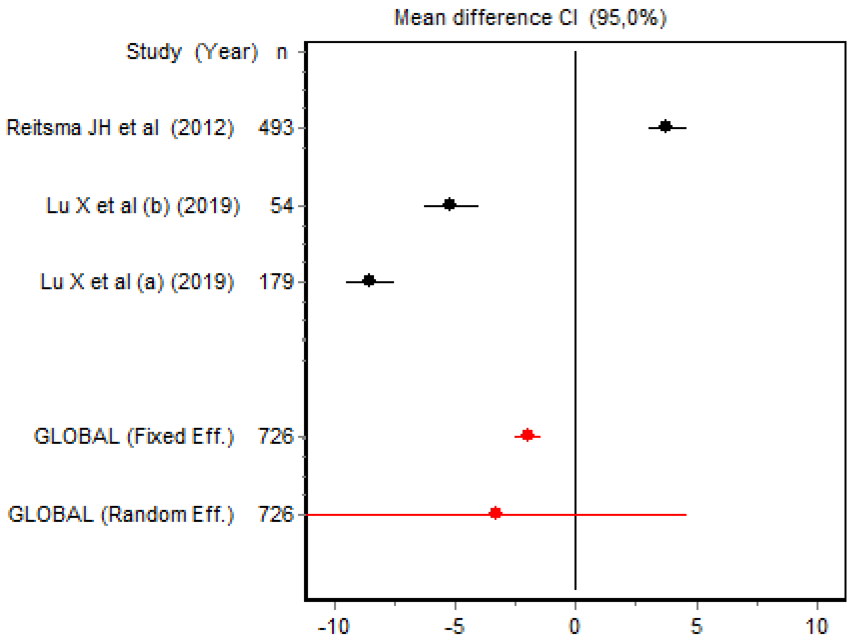

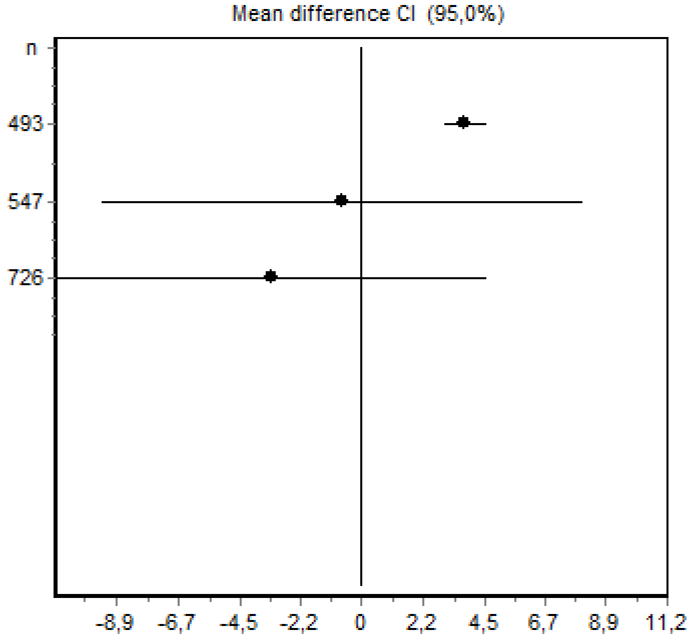


PUBLICATION BIAS

Begg test

| Z statistic | p-value |
| --- | --- |
| 0,0000 | 1,0000 |

Egger test

| t statistic | df | p-value |
| --- | --- | --- |
| -1,3851 | 1 | 0,3981 |

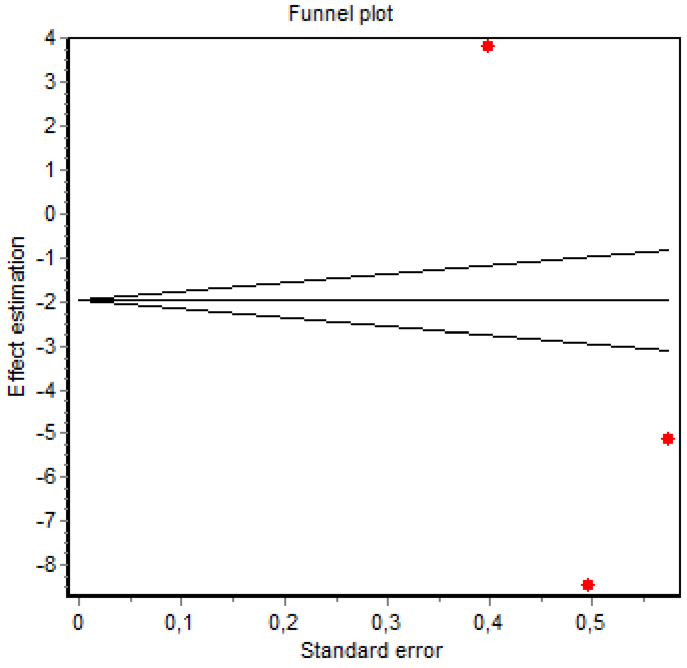


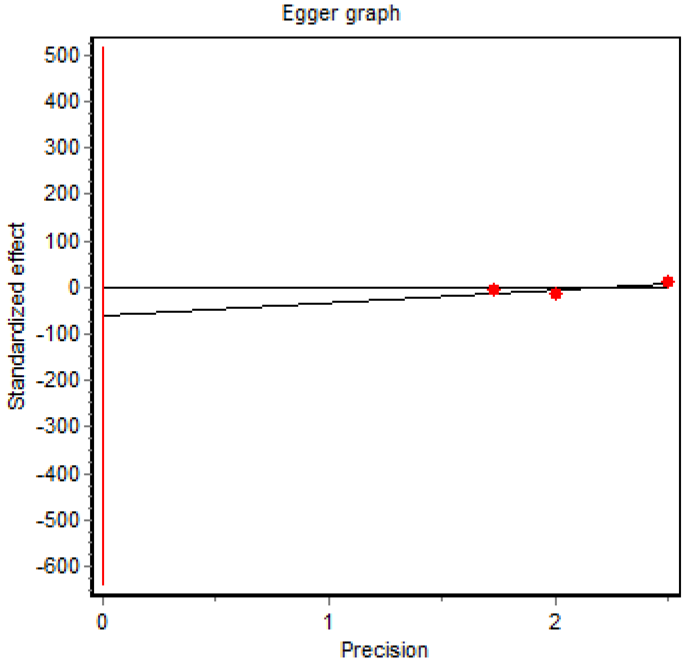


SENSITIVITY ANALYSIS

RANDOM EFFECTS MODEL

CI(95,0%)

Omitted study Year n d Lower limit Upper limit Relative change (%)

| | | | | | | |
|---|---|---|---|---|---|---|
| Reitsma JH et al | 2012 | 233 | -6,8493 | -10,1126 | -3,5861 | 108,30 |
| Lu X et al (b) | 2019 | 672 | -2,3510 | -14,3992 | 9,6972 | -28,50 |
| Lu X et al (a) | 2019 | 547 | -0,6800 | -9,4648 | 8,1048 | -79,32 |
| GLOBAL | | 726 | -3,2883 | -11,1758 | 4,5992 | |

Influence graph

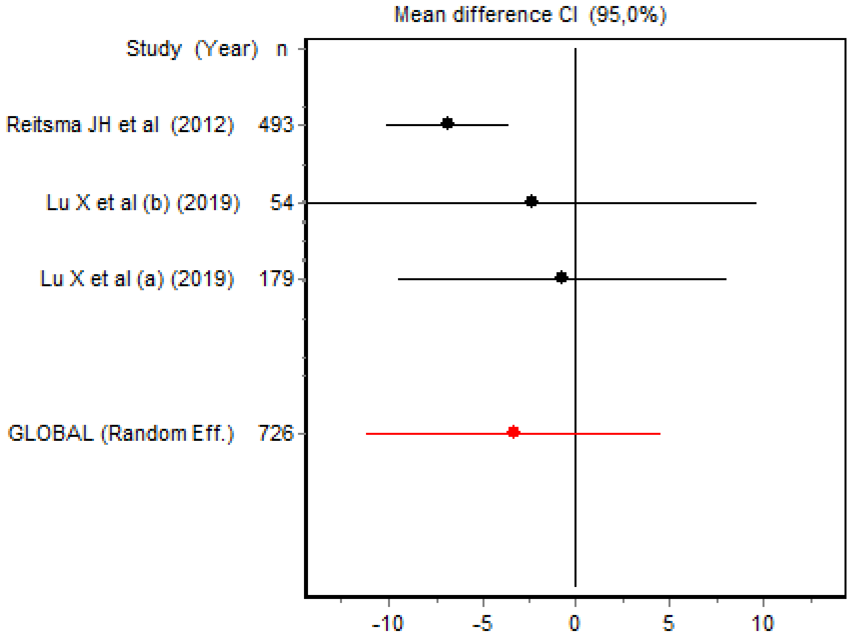

## -- Down Syndrome: LOWER MAXILLA (SNB)--

Confidence level: 95,0%
Number of studies: 2
Sort by: Year
Sorting orientation: Ascending

HETEROGENEITY

Dersimonian and Laird's heterogeneity test

| Q statistic (Chi-square) | df | p-value |
|---|---|---|
| 148,2277 | 1 | 0,0000 |

| Heterogeneity statistics | Estimator |
|---|---|
| Variance between studies | 31,3621 |
| Variance within studies | 0,1672 |
| Coefficient RI | 0,9947 |
| Variation coeff. between studies | 1,4152 |

Coefficient RI (Proportion of total variance due to the variance between studies)

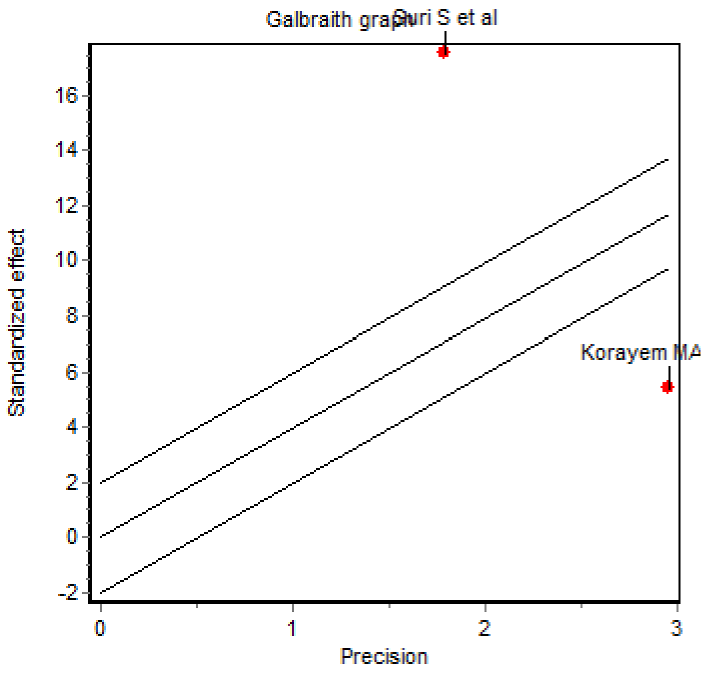


INDIVIDUAL AND COMBINED RESULTS

| Study | Year | n | d | CI(95,0%) | Weights(%) Fixed eff. | Weights(%) Random eff. |
|---|---|---|---|---|---|---|

| | | | | | | | |
|---|---|---|---|---|---|---|---|
| Suri S et al | 2010 | 179 | 9,7733 | 8,6789 | 10,8678 | 26,8117 | 49,8436 |
| Korayem MA et al | 2014 | 54 | 1,8266 | 1,1642 | 2,4890 | 73,1883 | 50,1564 |
| Fixed effects | | 233 | 3,9573 | 3,3906 | 4,5240 | | |
| Random effects | | 233 | 5,7875 | -2,0000 | 13,5751 | | |

FOREST PLOT CUMULATIVE META-ANALYSIS(Random effects)

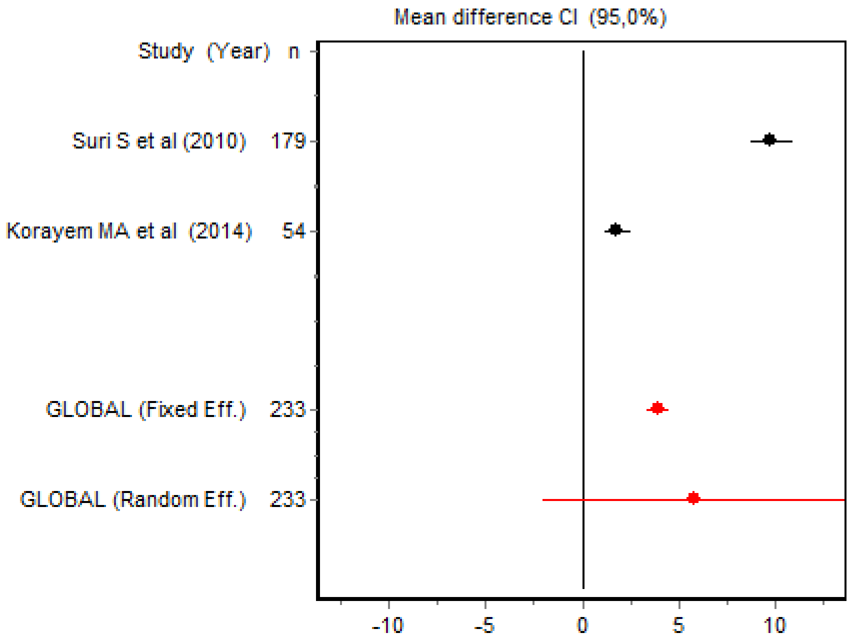

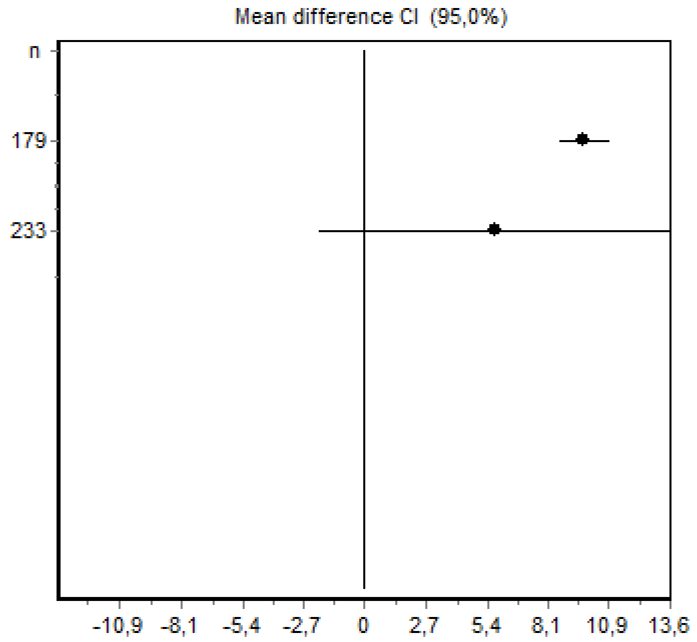


PUBLICATION BIAS

Begg test

| Z statistic | p-value |
| --- | --- |
| 0,0000 | 1,0000 |

## -- Klinefelter Syndrome: LOWER MAXILLA (SNPg)--

```
  Confidence level:  95,0%
 Number of studies: 3
           Sort by: Year
Sorting orientation: Ascending
```

HETEROGENEITY

Dersimonian and Laird's heterogeneity test

| Q statistic (Chi-square) | df | p-value |
|---|---|---|
| 392,4555 | 2 | 0,0000 |

| Heterogeneity statistics | Estimator |
|---|---|
| Variance between studies | 1063,4161 |
| Variance within studies | 0,4393 |
| Coefficient RI | 0,9996 |

(Proportion of total variance due to the variance between studies)

| | |
|---|---|
| Variation coeff. between studies | 6,6101 |

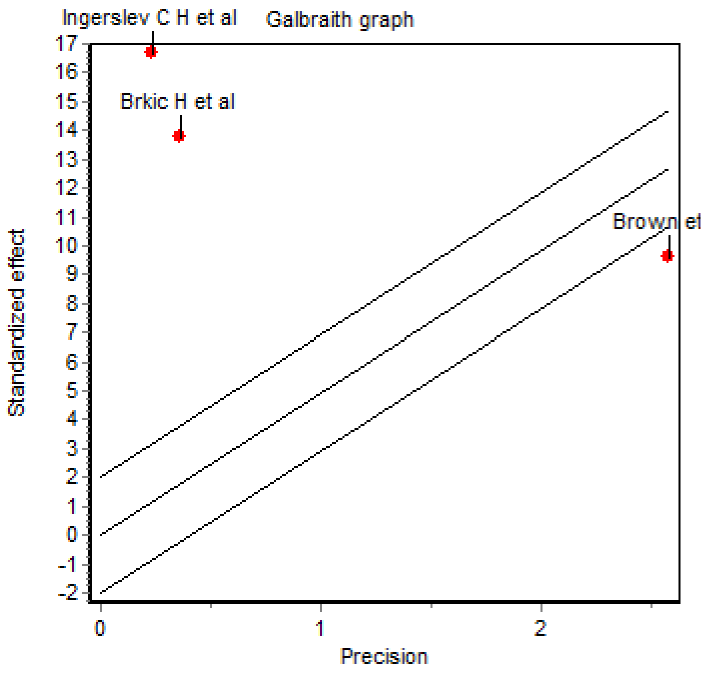


INDIVIDUAL AND COMBINED RESULTS

| Study | Year | n | d | CI(95,0%) | Weights(%) Fixed eff. | Weights(%) Random eff. |
|---|---|---|---|---|---|---|

| | | | | | | | |
|---|---|---|---|---|---|---|---|
| Ingerslev C H et al | 1978 | 139 | 70,6101 | 62,3013 | 78,9189 | 0,8148 | 33,0419 |
| Brown et al | 1993 | 73 | 3,7293 | 2,9688 | 4,4899 | 97,2521 | 33,5955 |
| Brkic H et al | 1994 | 95 | 37,8227 | 32,4285 | 43,2168 | 1,9331 | 33,3626 |
| Fixed effects | | 307 | 4,9333 | 4,1834 | 5,6833 | | |
| Random effects | | 307 | 37,2024 | 0,1539 | 74,2510 | | |

FOREST PLOT CUMULATIVE META-ANALYSIS(Random effects)

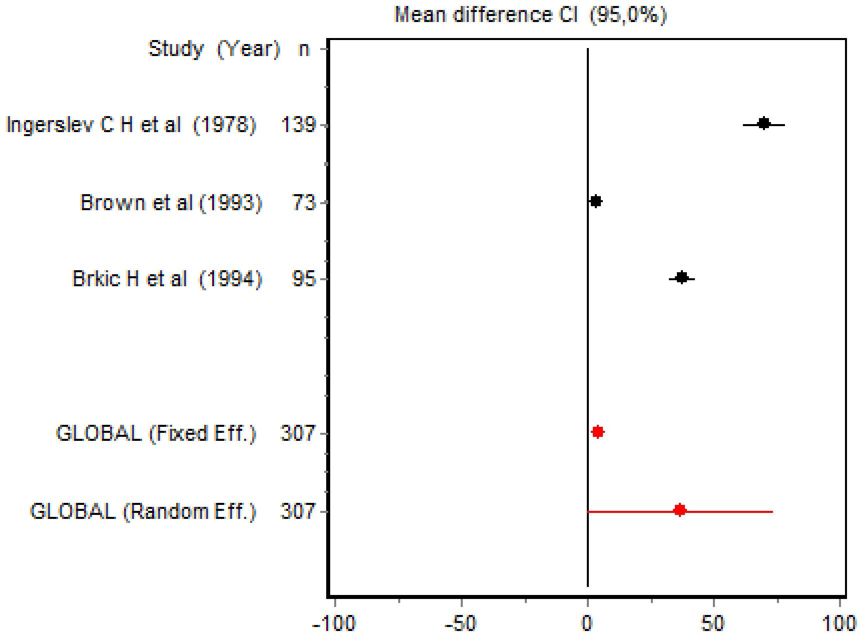

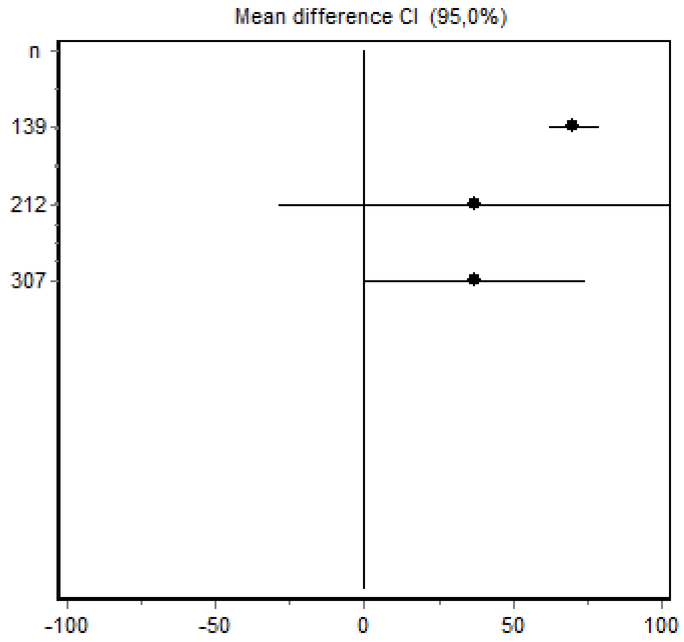


PUBLICATION BIAS

Begg test

| Z statistic | p-value |
|---|---|
| 1,0445 | 0,2963 |

Egger test

| t statistic | df | p-value |
|---|---|---|
| 10,7389 | 1 | 0,0591 |

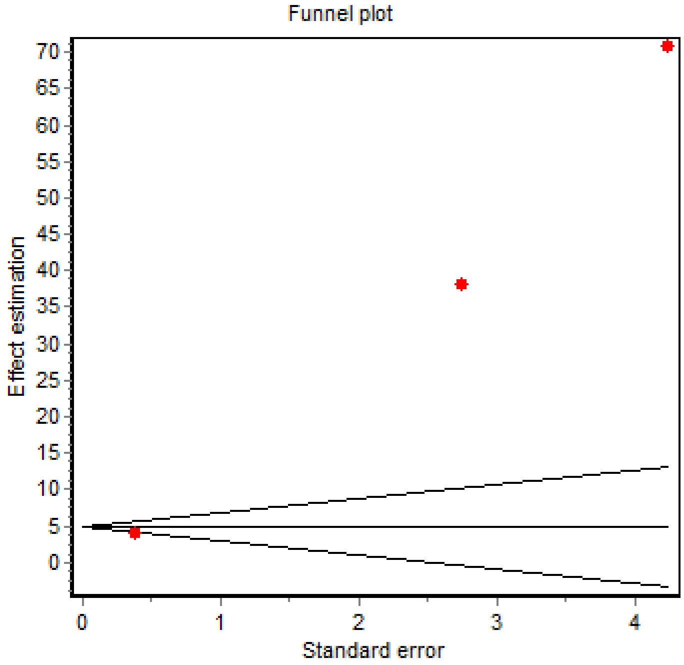


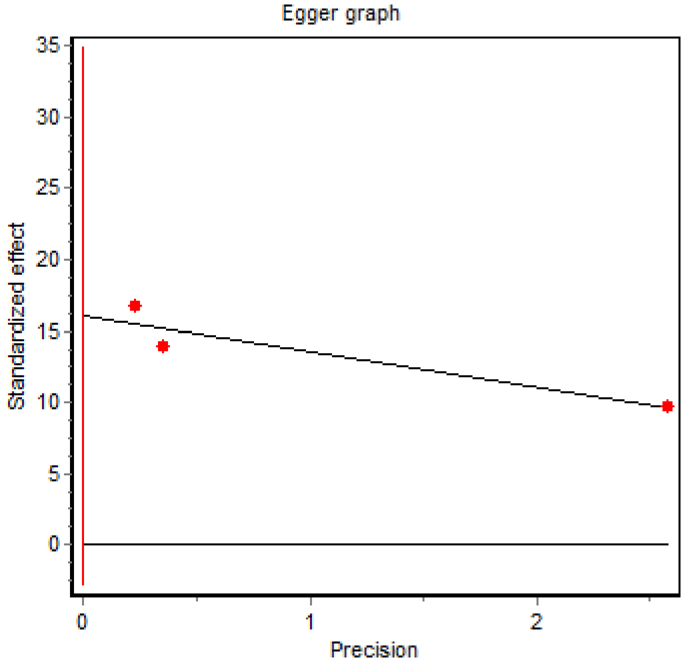


SENSITIVITY ANALYSIS

RANDOM EFFECTS MODEL

CI(95,0%)
Omitted study Year n d Lower limit Upper limit Relative change (%)

| | | | | | | |
|---|---|---|---|---|---|---|
| Ingerslev C H et al | 1978 | 168 | 20,6671 | -12,7430 | 54,0773 | -44,45 |
| Brown et al | 1993 | 234 | 54,0578 | 21,9282 | 86,1874 | 45,31 |
| Brkic H et al | 1994 | 212 | 37,0365 | -28,5049 | 102,5779 | -0,45 |
| GLOBAL | | 307 | 37,2024 | 0,1539 | 74,2510 | |

Influence graph

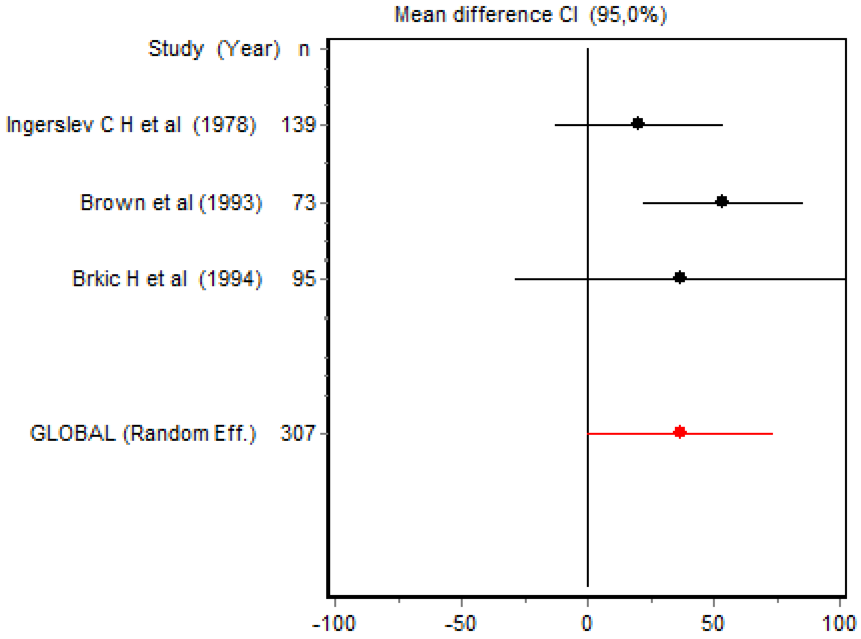

## -- X Linked Hypohidrotic Ectoderrmal dysplasia: LOWER MAXILLA (SNPg)--

Confidence level: 95,0%
Number of studies: 3
Sort by: Year
Sorting orientation: Ascending

HETEROGENEITY

Dersimonian and Laird's heterogeneity test

| Q statistic (Chi-square) | df | p-value |
|---|---|---|
| 195,9904 | 2 | 0,0000 |

| Heterogeneity statistics | Estimator |
|---|---|
| Variance between studies | 9,4804 |
| Variance within studies | 0,0883 |
| Coefficient RI | 0,9908 |

(Proportion of total variance due to the variance between studies)

| | |
|---|---|
| Variation coeff. between studies | 0,7826 |

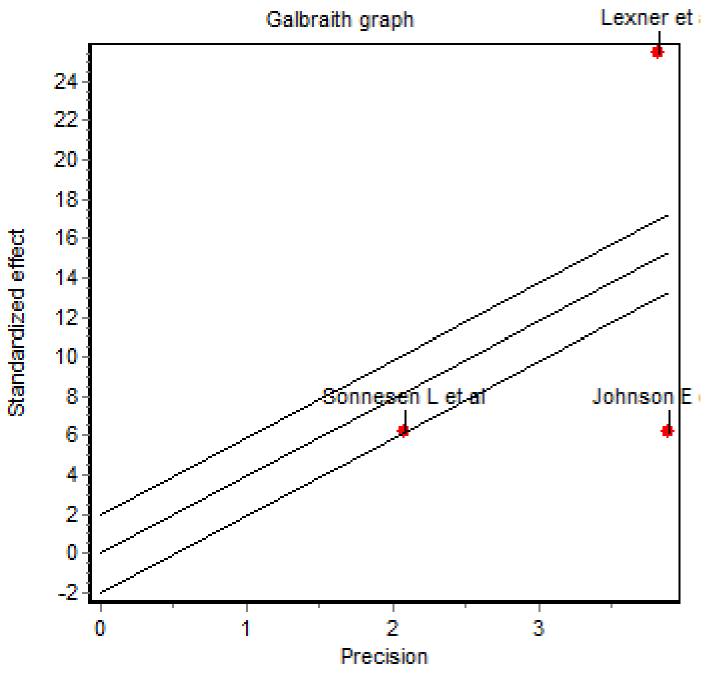


INDIVIDUAL AND COMBINED RESULTS

| Study | Year | n | d | CI(95,0%) | Weights(%) Fixed eff. | Weights(%) Random eff. |
|---|---|---|---|---|---|---|

| | | | | | | | |
|---|---|---|---|---|---|---|---|
| --------------------- | ---- | ------ | ------ | ------ | ------ | ------- | ------- |
| Johnson E et al | 2002 | 148 | 1,5786 | 1,0741 | 2,0830 | 44,4651 | 33,5271 |
| Lexner et al | 2007 | 512 | 6,6623 | 6,1484 | 7,1761 | 42,8459 | 33,5183 |
| Sonnesen L et al | 2017 | 37 | 2,9795 | 2,0353 | 3,9237 | 12,6891 | 32,9546 |
| --------------------- | ---- | ------ | ------ | ------ | ------ | ------- | ------- |
| Fixed effects | | 697 | 3,9345 | 3,5981 | 4,2708 | | |
| Random effects | | 697 | 3,7442 | 0,2377 | 7,2507 | | |

FOREST PLOT CUMULATIVE META-ANALYSIS(Random effects)

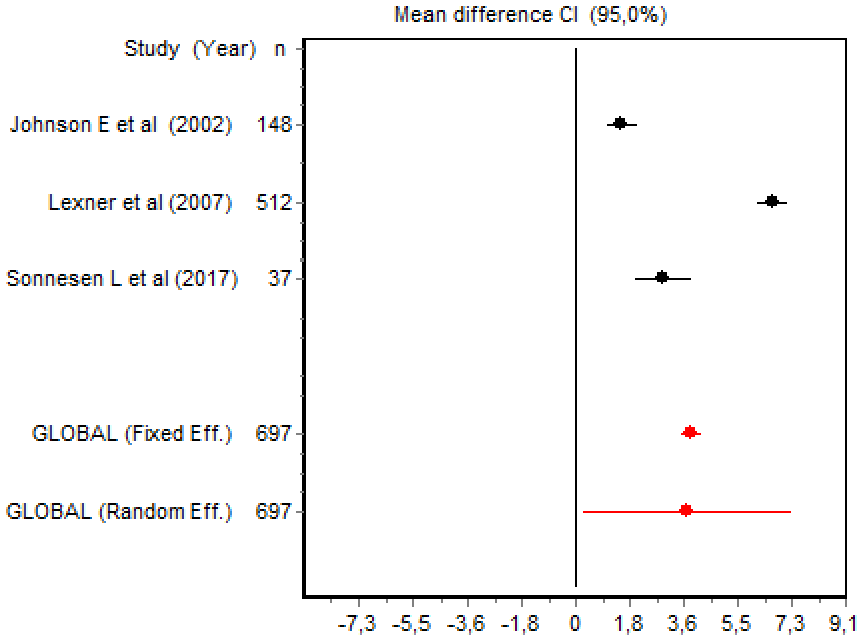

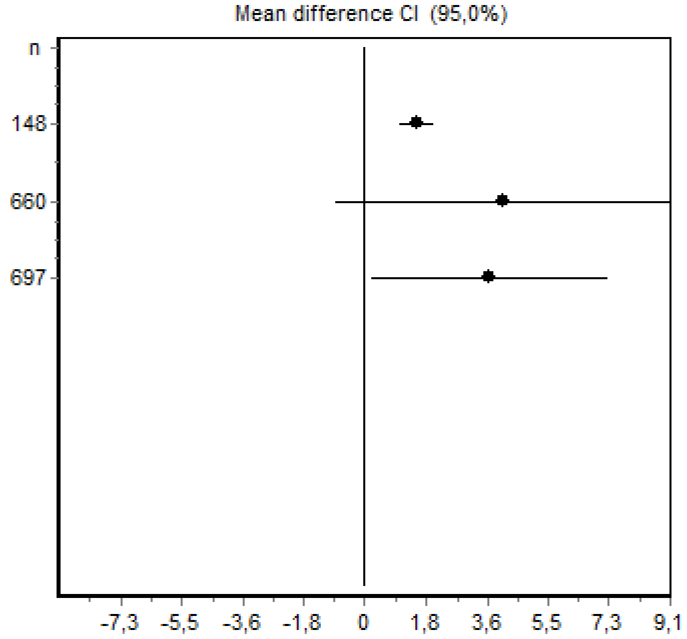


PUBLICATION BIAS

Begg test

| Z statistic | p-value |
|---|---|
| 0,0000 | 1,0000 |

Egger test

| t statistic | df | p-value |
|---|---|---|
| -0,1223 | 1 | 0,9225 |

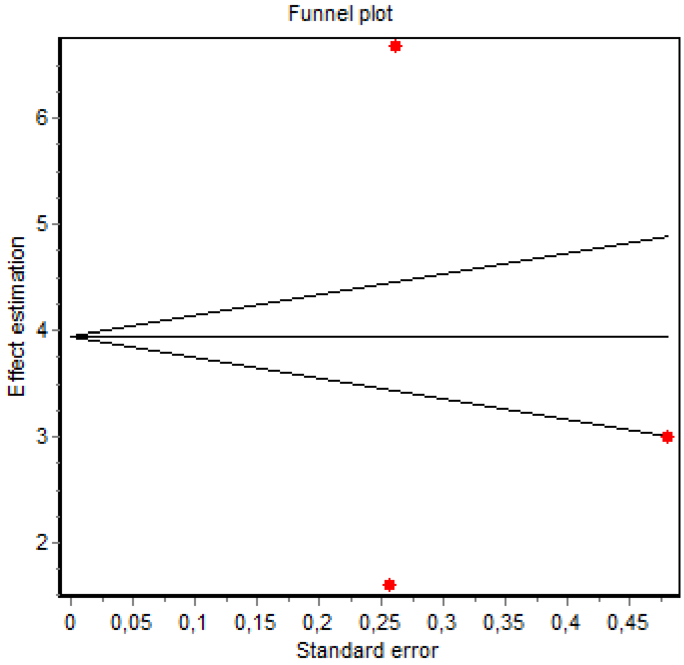


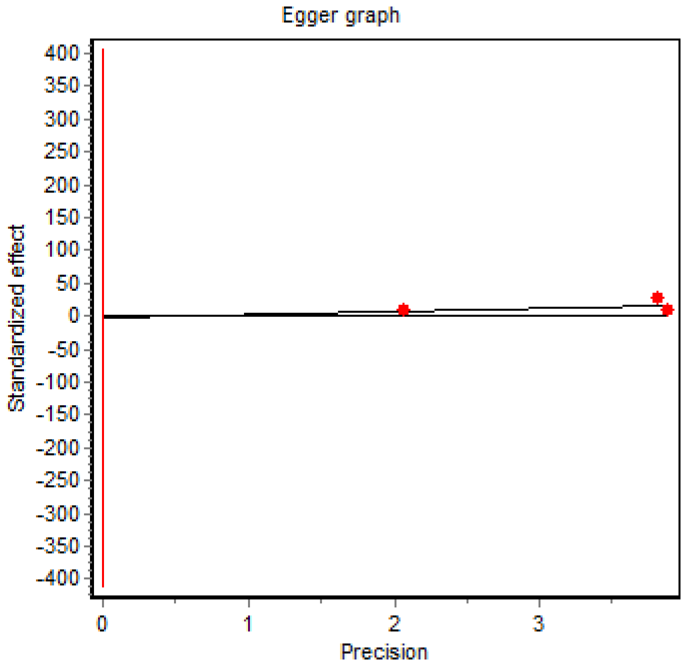


SENSITIVITY ANALYSIS

RANDOM EFFECTS MODEL

CI(95,0%)

Omitted study Year n d Lower limit Upper limit Relative change (%)

| | | | | | | |
|---|---|---|---|---|---|---|
| Johnson E et al | 2002 | 549 | 4,8431 | 1,2343 | 8,4519 | 29,35 |
| Lexner et al | 2007 | 185 | 2,2198 | 0,8518 | 3,5878 | -40,71 |
| Sonnesen L et al | 2017 | 660 | 4,1202 | -0,8618 | 9,1021 | 10,04 |
| GLOBAL | | 697 | 3,7442 | 0,2377 | 7,2507 | |

Influence graph

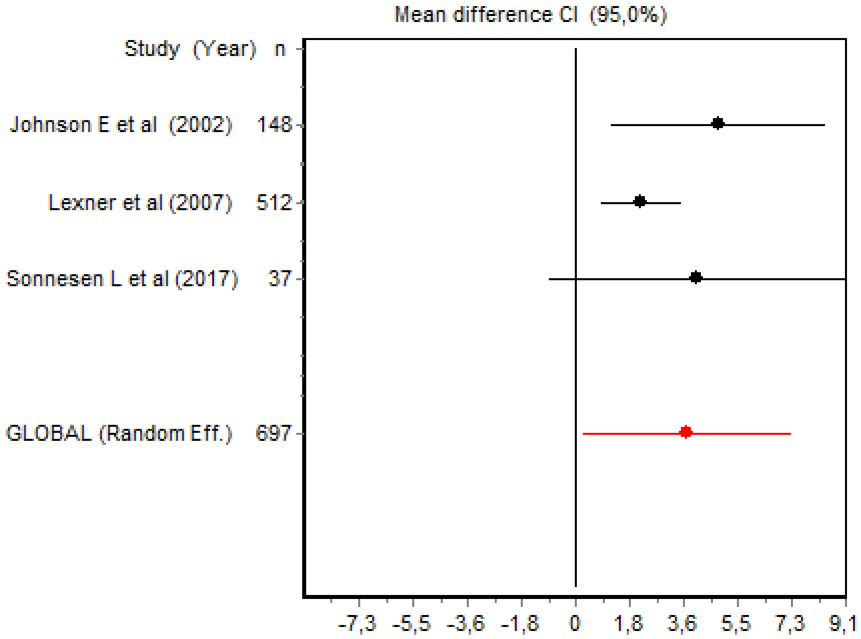

Supplement: Supplementary file 8 [file 41390_2023_2907_MOESM8_ESM.pdf]
