# Supplementary figures and images for: Craniofacial syndromes and class III phenotype: common genotype fingerprints? A scoping review and meta-analysis
Source: Pediatr Res. 2024 Feb 12;95(6):1455–75. doi: 10.1038/s41390-023-02907-5 (PMC11126392; doi:10.1038/s41390-023-02907-5)

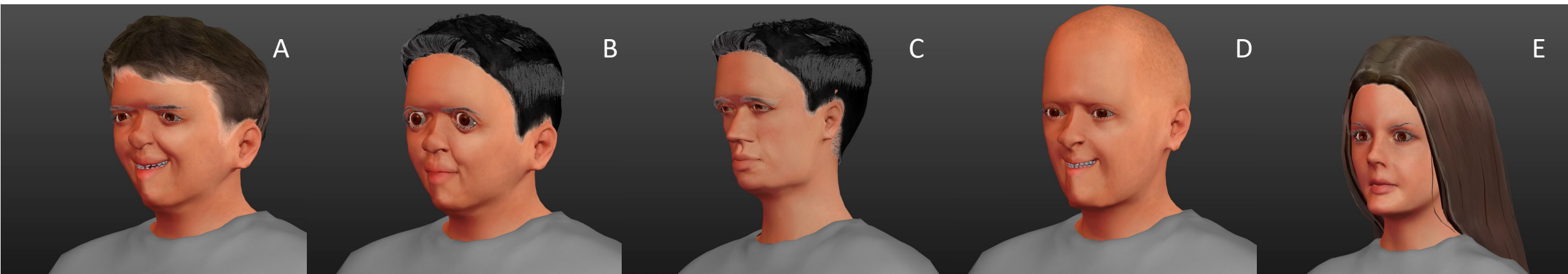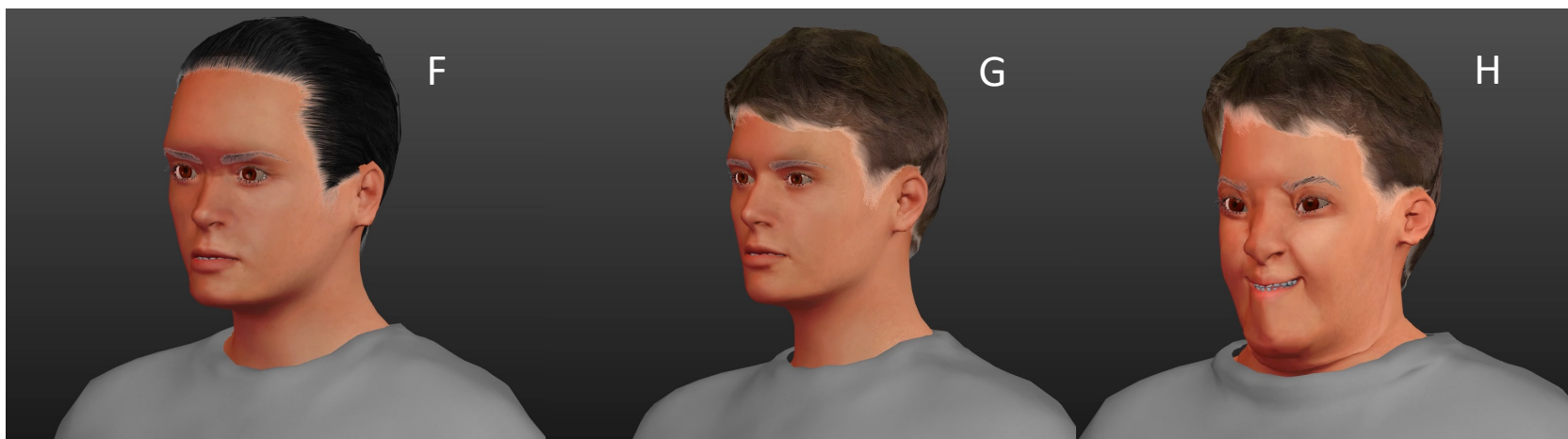

Supplement: Supplementary file 9 [file 41390_2023_2907_MOESM9_ESM.pdf]
